# International Union of Basic and Clinical Pharmacology CIII: Chemerin Receptors CMKLR1 (Chemerin_1_) and GPR1 (Chemerin_2_) Nomenclature, Pharmacology, and Function

**DOI:** 10.1124/pr.116.013177

**Published:** 2018-01

**Authors:** Amanda J. Kennedy, Anthony P. Davenport

**Affiliations:** Experimental Medicine and Immunotherapeutics, University of Cambridge, Centre for Clinical Investigation, Addenbrooke’s Hospital, Cambridge, United Kingdom

## Abstract

Chemerin, a chemoattractant protein and adipokine, has been identified as the endogenous ligand for a G protein–coupled receptor encoded by the gene *CMKLR1* (also known as ChemR23), and as a consequence the receptor protein was renamed the chemerin receptor in 2013. Since then, chemerin has been identified as the endogenous ligand for a second G protein–coupled receptor, encoded by the gene *GPR1*. Therefore, the International Union of Basic and Clinical Pharmacology Committee on Receptor Nomenclature and Drug Classification recommends that the official name of the receptor protein for chemokine-like receptor 1 (CMKLR1) is chemerin receptor 1, and G protein–coupled receptor 1 is chemerin receptor 2 to follow the convention of naming the receptor protein after the endogenous ligand. Chemerin receptor 1 and chemerin receptor 2 can be abbreviated to Chemerin_1_ and Chemerin_2_, respectively. Chemerin requires C-terminal processing for activity, and human chemerin21–157 is reported to be the most active form, with peptide fragments derived from the C terminus biologically active at both receptors. Small-molecule antagonist, CCX832, selectively blocks CMKLR1, and resolvin E1 activation of CMKLR1 is discussed. Activation of both receptors by chemerin is via coupling to G_i/o_, causing inhibition of adenylyl cyclase and increased Ca^2+^ flux. Receptors and ligand are widely expressed in humans, rats, and mice, and both receptors share ∼80% identity across these species. *CMKLR1* knockout mice highlight the role of this receptor in inflammation and obesity, and similarly, *GPR1* knockout mice exhibit glucose intolerance. In addition, the chemerin receptors have been implicated in cardiovascular disease, cancer, steroidogenesis, human immunodeficiency virus replication, and neurogenerative disease.

## I. Introduction

**Fig. 1.**
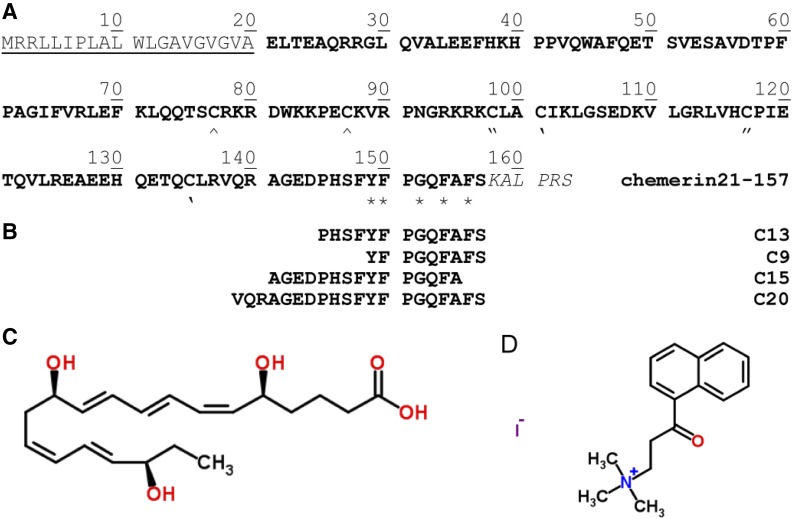
Agonists and antagonists of the chemerin receptors. (A) The 163-amino-acid sequence of chemerin (Uniprot: Q99969). The signal peptide is underlined; the cysteine residues involved in three disulfide bonds are marked ^, “, and ‘ in pairs. The residues marked with * were found to be important for binding to chemerin receptor 1 by alanine screening. (B) Amino acids corresponding to C9, C13, C15, and C20 synthetic peptides that activate downstream signalling at CMKLR1 and GPR1. Chemspider structures of (C) CMKLR1 agonist RvE1 and (D) CMKLR1 antagonist, α-NETO. NB. The structure of CCX832 is not publicly available.

Chemerin, the endogenous ligand of chemokine-like receptor 1 (CMKLR1) or ChemR23, was identified in 2003 as the product of the *RARRES2* gene (Meder et al., 2003; Wittamer et al., 2003). In 1997, the RARRES2 gene was first identified as a novel retinoid-responsive gene in psoriatic skin lesions (Nagpal et al., 1997). As a consequence of its production in response to retinoid substances, the gene product was initially christened as tazarotene-induced gene 2 (TIG2) or retinoic acid receptor responder 2 protein (RARRES2). The human gene translates into a 163-amino-acid protein (mol. wt. 18,618 Da), made up of a 20-amino-acid hydrophobic N-terminal signal peptide, an intervening 137-amino-acid cystatin-fold containing domain, and a six-amino-acid C-terminal prosegment (Fig. 1A). Amino acids 21–157, corresponding to the 137-amino-acid intervening region, were found to be the active part of the protein and subsequently named chemerin (Wittamer et al., 2003). Prochemerin, the 143-amino-acid precursor protein (21–163), released following cleavage of the signal peptide, circulates in the plasma and has low biologic activity; it needs to be further processed at the C terminus to give the active form (Meder et al., 2003; Wittamer et al., 2003; Zabel et al., 2005a,b; Cash et al., 2008; Du et al., 2009; Ernst and Sinal, 2010). More detailed reviews are listed in Table 1. Human chemerin21**–**157 is reported to be the most active form; removal of one amino acid (chemerin21**–**156) resulted in a sixfold drop in potency, whereas the addition of one or removal of two or three amino acids strongly affected potency, with no response seen up to 10 *μ*M (Wittamer et al., 2004). The C terminus is therefore very important for function at CMKLR1, exemplified further by synthetic C-terminal fragments of human chemerin: C9 (or chemerin-9), chemerin149**–**157; C13, chemerin145**–**157 (Wittamer et al., 2004); and C20, chemerin138**–**157 (Li et al., 2014a), possessing biologic activity (Fig. 1C). It is not yet known whether these short peptides are generated endogenously.

**TABLE 1:** Detailed reviews

Focus of the review	Reference							
Chemerin activation	Zabel et al., 2006; Ernst and Sinal, 2010; Mattern et al., 2014							
Chemerin receptors	Yoshimura and Oppenheim, 2011							
Function of chemerin	Mattern et al., 2014; Ferland and Watts, 2015; Mariani and Roncucci, 2015; Fatima et al., 2014; Zabel et al., 2014; Bondue et al., 2011b							
General overview	Ernst and Sinal, 2010; Fatima et al., 2014; Mattern et al., 2014							
Inflammation	Zabel et al., 2014; Mariani and Roncucci, 2015							
Obesity	Roman et al., 2012							
Cardiovascular disease	Ferland and Watts, 2015							
Cancer	Mariani and Roncucci, 2015							

In addition to activating CMKLR1, human chemerin21**–**157, C9, and C13 have also been found to activate orphan receptor, G protein**–**coupled receptor 1 (GPR1). This pairing was initially discovered due to the high sequence identity between GPR1 and CMKLR1 (Barnea et al., 2008) and has subsequently been independently confirmed (Southern et al., 2013; Rourke et al., 2014; Kennedy et al., 2016).

Chemerin has also been shown to bind to chemokine (C-C motif) receptor-like 2, but it does not activate any downstream signaling pathways or internalization (Zabel et al., 2008). Chemokine (C-C motif) receptor-like 2 is therefore designated an atypical chemokine receptor (Bachelerie et al., 2015) and is not covered in this review of signaling receptors. For more information, please see the review by Yoshimura and Oppenheim (2011).

The initial interest in the chemerin system was focused on its role in inflammation and chemotaxis of immune cells following its discovery in psoriasis. However, in 2007, there was a fundamental shift in the understanding of chemerin biology when its function as an adipokine was identified. More recently, in connection with its roles in inflammation, obesity, and metabolic syndrome, a potential role in the cardiovascular system is being considered, as well as roles in reproductive biology. This review will focus on the pharmacology of the signaling chemerin receptors; for more detailed information on function, the reviews listed in Table 1 should be consulted.

In accordance with the standard International Union of Basic and Clinical Pharmacology Committee on Receptor Nomenclature and Drug Classification (NC-IUPHAR) rules of nomenclature, which state that a receptor is named after its endogenous agonist (Vanhoutte et al., 1996), the CMKLR1 receptor was renamed the chemerin receptor in 2013 (Davenport et al., 2013). Following the identification of GPR1 as a second signaling chemerin receptor, we therefore propose that, at the protein level, CMKLR1 is referred to as chemerin receptor 1 and GPR1 is referred to as chemerin receptor 2. We also suggest abbreviating chemerin receptor 1 and chemerin receptor 2 to Chemerin_1_ and Chemerin_2_, respectively. When referring to chemerin as an agonist for these receptors, it is imperative to state the number of the corresponding amino acids and which species chemerin is derived from, for example, human chemerin21–157, in which the first number represents the first amino acid from the 163-amino-acid prochemerin sequence, and the second number represents the last. Note the numbering of amino acids in the mouse sequence corresponding to the same amino acid of the human sequence differs by one due to the mouse sequence being one amino acid shorter. Smaller fragments, such as C9, C13, and C20, can be abbreviated, but for clarity it is still necessary to state the species and corresponding residues on first use. Names of genes should be italicized and, consistent with the Human Genome Organization, *CMKLR1* refers to chemerin receptor 1, *GPR1* refers to chemerin receptor 2, and *RARRES2* refers to chemerin, with lower case letters used for nonhuman species (Table 2).

**TABLE 2:** Nomenclature of chemerin and its receptors

Chemerin	Species	Gene	Protein
Receptor 1	Human	*CMKLR1*	Chemerin receptor 1
	Nonhuman	*cmklr1*	Chemerin receptor 1
Receptor 2	Human	*GPR1*	Chemerin receptor 2
	Nonhuman	*gpr1*	Chemerin receptor 2
Peptide	Human	*RARRES2*	Chemerin
	Nonhuman	*rarres2*	Chemerin

## II. Chemokine-Like Receptor 1 Designated as Chemerin Receptor 1

**Fig. 2.**
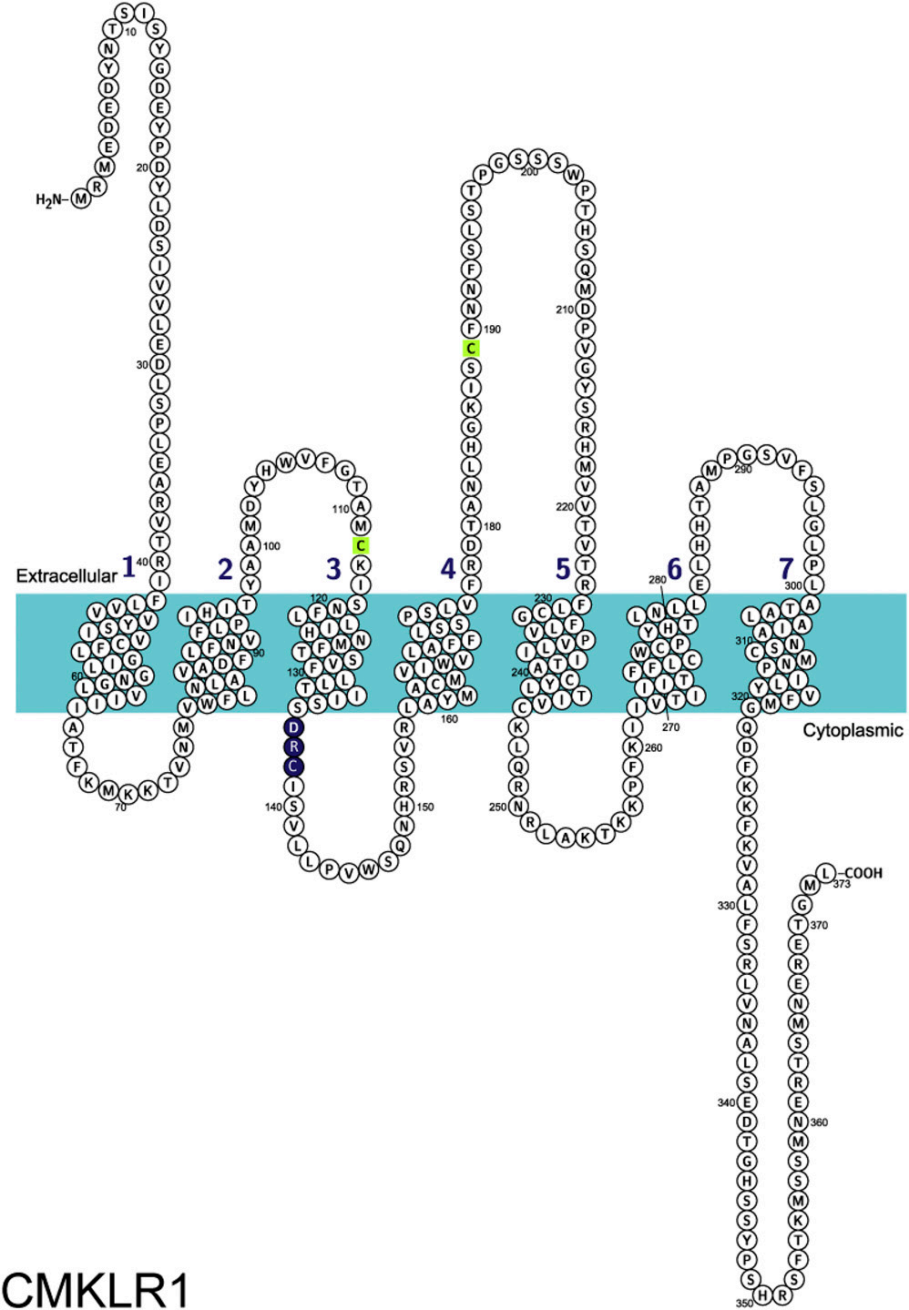
Amino acid sequence of chemerin receptor 1: Cys^112^ andCys^189^ (green) are predicted to form a disulfide bond based on sequence similarity, and the G protein–binding motif is shown in blue. Figure made using UniProt (Q99788) and Protter (Omasits et al., 2014).

**Fig. 3.**
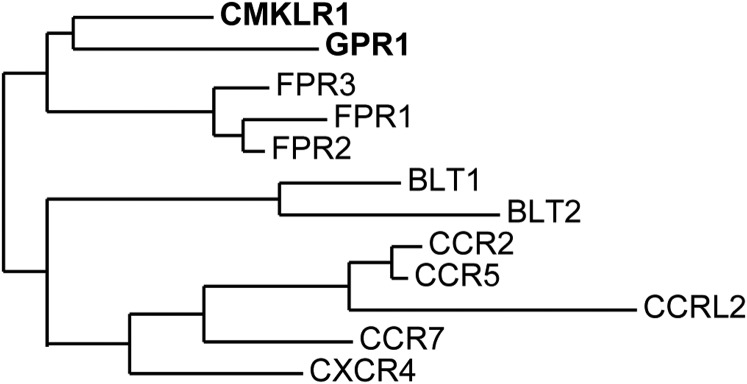
Schematic representation of the structural similarities between chemerin receptors, CMKLR1 and GPR1, and other chemoattractant receptors. Sequences for the receptors were aligned to generate the phylogenetic tree (http://www.phylogeny.fr/). The receptors include chemokine receptors (CCR2, CCR5, CCR7, and CXCR4), leukotriene B receptors (BLT1 and BLT2), and formyl peptide receptors (FRP1, FRP2, and FRP3).

Gantz et al. (1996) cloned a novel human gene, encoding the orphan receptor CMKLR1, which had sequence and structural homology with a seven-transmembrane G protein–coupled receptor (GPCR) (Fig. 2). Samson et al. (1998) cloned what they thought was a novel human gene encoding the same GPCR, which they named ChemR23. The receptor is structurally related to receptors for chemokines and other chemoattractant molecules such as leukotriene B4 and resolvin D1 (Fig. 3). In 2003, two independent groups (Meder et al., 2003; Wittamer et al., 2003) identified a novel chemoattractant in human biologic fluids, the product of the *RARRES2* gene, as the endogenous ligand for CMKLR1. Wittamer et al. (2003) renamed the gene product chemerin, and reported that the active isoform in ascitic fluids was human chemerin21–157, whereas Meder et al. (2003) found human chemerin21–154 in hemofiltrate. This pairing was independently confirmed when Zabel et al. (2005b) identified that human chemerin21–155 from serum was active at the CMKLR1 receptor. C9 has been reported as the minimum length of fragment needed to bind and activate chemerin receptor 1 (Wittamer et al., 2004), retaining a nanomolar potency in calcium assays. However, further studies into the activation of different signaling pathways suggest that C9 does not mimic fully the actions of chemerin21–157. C9 exhibits bias, and it is significantly less potent at activating *β*-arrestin recruitment compared with chemerin21–157 (Kennedy et al., 2016; see *Section VIII. A. Signaling Pathways Activated by Chemerin Receptor 1*). The protein of the *CMKLR1* gene was renamed the chemerin receptor (Davenport et al., 2013) following confirmation of chemerin as its ligand. A second chemerin receptor has now been identified; therefore, CMKLR1 should be designated chemerin receptor 1 for ligand chemerin (Table 3).

**TABLE 3:** Classification of chemerin receptor 1

Receptor Structure, Pharmacology, and Distribution	Receptor Amino Acid Sequences, Pharmacological Parameters, Tissue Distribution	References
Previous Names	CMKLR1, ChemR23, ChemerinR, GPCR27, DEZ, RVER1, TIG2 receptor	
Structural information	7TM	
Humans	373 aa (UniProt Q99788) chr. 12q24.1 (Entrez 1240)	
Rats	371 aa (UniProt O35786) chr. 12q16 (Entrez 60669)	
Mice	371 aa (UniProt P97468) chr. 5F (Entrez 14747)	
Functional assays	CHO cells transfected with CMKLR1	Wittamer et al., 2003; Barnea et al., 2008
	Chemotaxis migration assays	Wittamer et al., 2003; Vermi et al., 2005; Zabel et al., 2005b; Albanesi et al., 2009
	In vitro pharmacology using isolated human vessels	Kennedy et al., 2016
	In vitro pharmacology using isolated rat aorta	Watts et al., 2013
Endogenous agonists	Human chemerin(21–157) (pEC_50_ = 9.37 ± 0.05)	Wittamer et al., 2003
	RvE1 (pEC_50_ = 9.37 ± 0.05)	Arita et al., 2005
Agonists	C9 [chemerin(149–157)]	Wittamer et al., 2009
	C13 [chemerin(145–157)]	Wittamer et al., 2003
	C15 [chemerin(141–155)]	Cash et al., 2008
	C19 [chemerin(139–157)]	Wittamer et al., 2004
	C20 [chemerin(138–157)]	Li et al., 2014a
Selective antagonist	CCX832 (pIC_50_ = 8.34 ± 0.04)	Watts et al., 2013; Kennedy et al., 2016
Radioligands	[^125^I]-C9 (K_D_ = 4.9 nM)	Barnea et al., 2008; Kennedy et al., 2016
	Human [^125^I]-chemerin(21–157) (K_D_ = 0.88 nM)	De Henau et al., 2016
	Human [^125^I]Tyr-[Phe^149^]-chemerin146–157 (K_D_ = 22 nM)	Wittamer et al., 2003
	[^3^H]RvE1 (K_D_ = 11.3 ± 5.4)	Arita et al., 2005, 2007
	Mouse [^125^I]-chemerin(21–148) (EC_50_ = 1.6 nM)	Zabel et al., 2008; Bondue et al., 2012
Transduction mechanisms	Coupled to G_i/o_ proteins	Wittamer et al., 2003; Cash et al., 2008; Kennedy et al., 2016
		
Receptor distribution		
Humans	RT-PCR showed highest expression of CMKLR1 mRNA in the skin, adipose tissue, spleen, lymph nodes, and lung	Wittamer et al., 2003; Roh et al., 2007
	Immunostaining and FACS analysis confirmed high expression on dendritic cells, monocytes, and macrophages	Wittamer et al., 2003; Vermi et al., 2005; Zabel et al., 2005b; Herová et al., 2015
	CMKLR1 protein expression was found on smooth muscle cells of human vessels by immunohistochemistry	Kostopoulos et al., 2014
Mice	RT-PCR showed highest *CMKLR1* expression in white adipose tissue and the lung	Goralski et al., 2007
	CMKLR1 immunoreactivity was identified in adipocytes	Goralski et al., 2007
Rats	RT-PCR–detected CMKLR1 mRNA were in the reproductive system (testis and ovary)	Wang et al., 2012; Li et al., 2014b
	Western blot analysis and immunohistochemistry identified CMKLR1 expression in vascular endothelial cells, cardiomyocytes and the smooth muscle, and endothelium of aorta and mesenteric vessels	Watts et al., 2013; Zhao et al., 2013; Zhang et al., 2014
Tissue function	Chemotaxis of leukocytes; adipogenesis; antimicrobial agent; vasoconstrictor of saphenous vein and resistance arteries	Wittamer et al., 2003; Goralski et al., 2007; Cash et al., 2010; Banas et.al., 2013; Kennedy et al., 2016

aa, Amino acid; chr., chromosome; FACS, fluorescence-activated cell sorter; RT-PCR, reverse-transcription polymerase chain reaction.

**Fig. 4.**
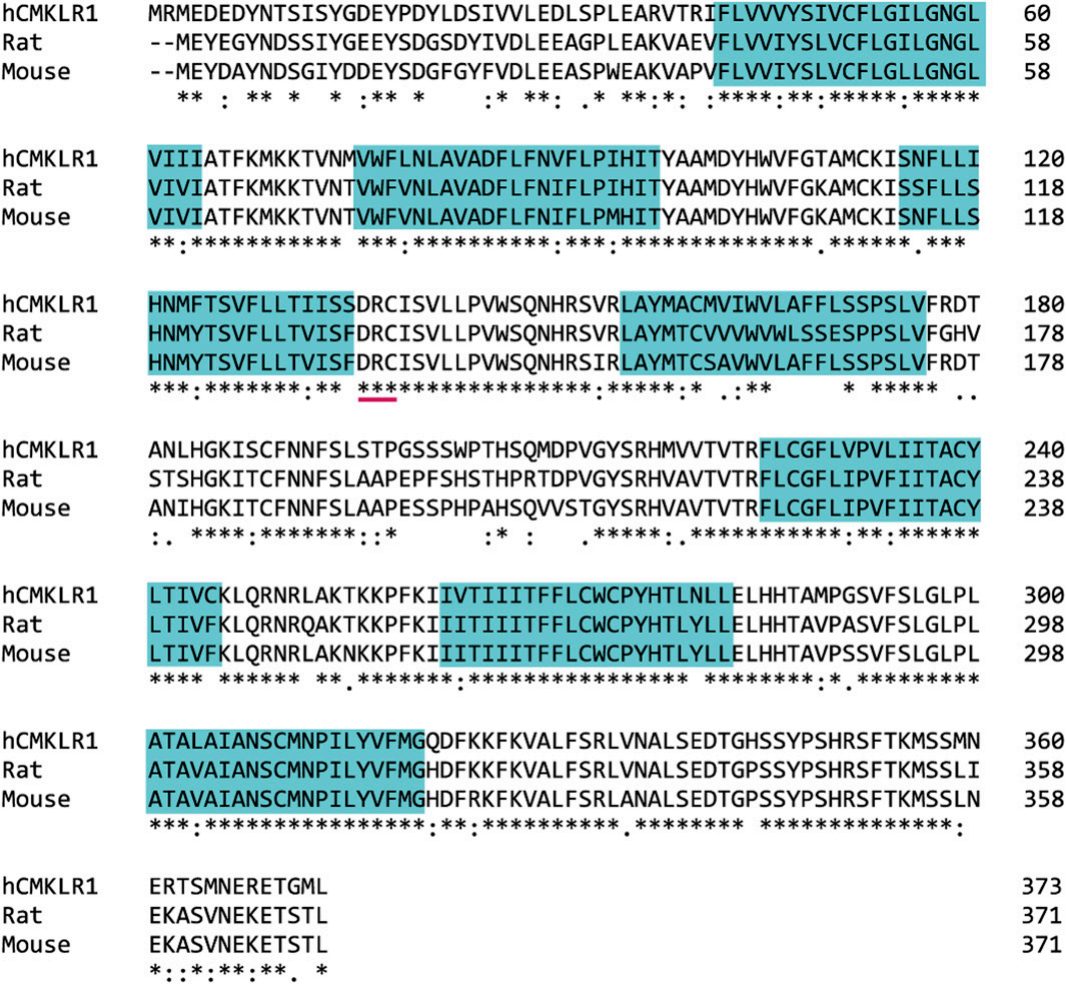
Clustal Omega (http://www.uniprot.org/align) sequence alignment of the human, rat, and mouse chemerin receptor 1. Identical amino acids in all species, “*”; conserved amino acid substitution, “:”; and semiconserved amino acid substitution, “.” Blue boxes highlight the transmembrane domains, and the pink line identifies the G protein–binding motif.

Chemerin receptor 1 is a class A GPCR coupled to G_i/o_, leading to inhibition of adenylyl cyclase and subsequent cAMP accumulation, intracellular calcium release, and phosphorylation of mitogen-activated protein kinases (MAPK) (Wittamer et al., 2003) (see *Section VIII. A. Signaling Pathways Activated by Chemerin Receptor 1*). Human chemerin receptor 1 shares 79% and 80% sequence identity with rat and mouse chemerin receptor 1, respectively (Fig. 4). The closest structural relative to chemerin receptor 1 is orphan receptor GPR1 (Fig. 3), which has since been paired with chemerin (see *Section III. G Protein–Coupled Receptor 1 Designated as Chemerin Receptor 2*).

## III. G Protein–Coupled Receptor 1 Designated as Chemerin Receptor 2

**Fig. 5.**
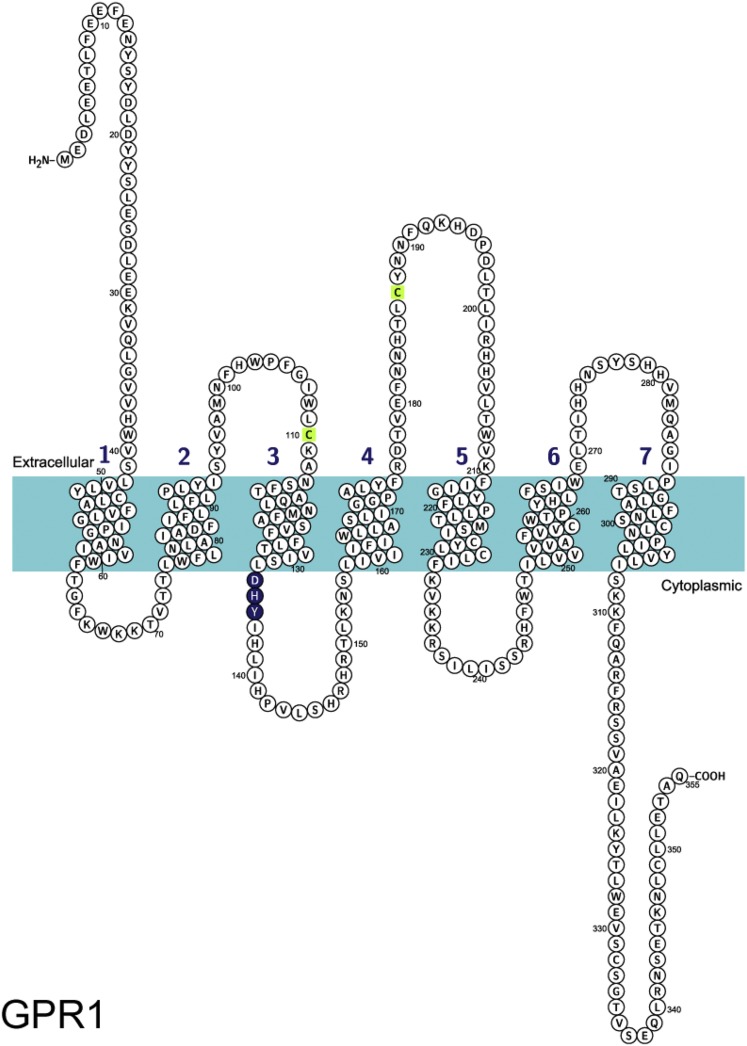
Amino acid sequence of chemerin receptor 2: Cys^110^ and Cys^187^ (green) are predicted to form a disulfide bond based on sequence similarity, and the G protein–binding motif is shown in blue. Figure made using UniProt (P46091) and Protter (Omasits et al., 2014).

**Fig. 6.**
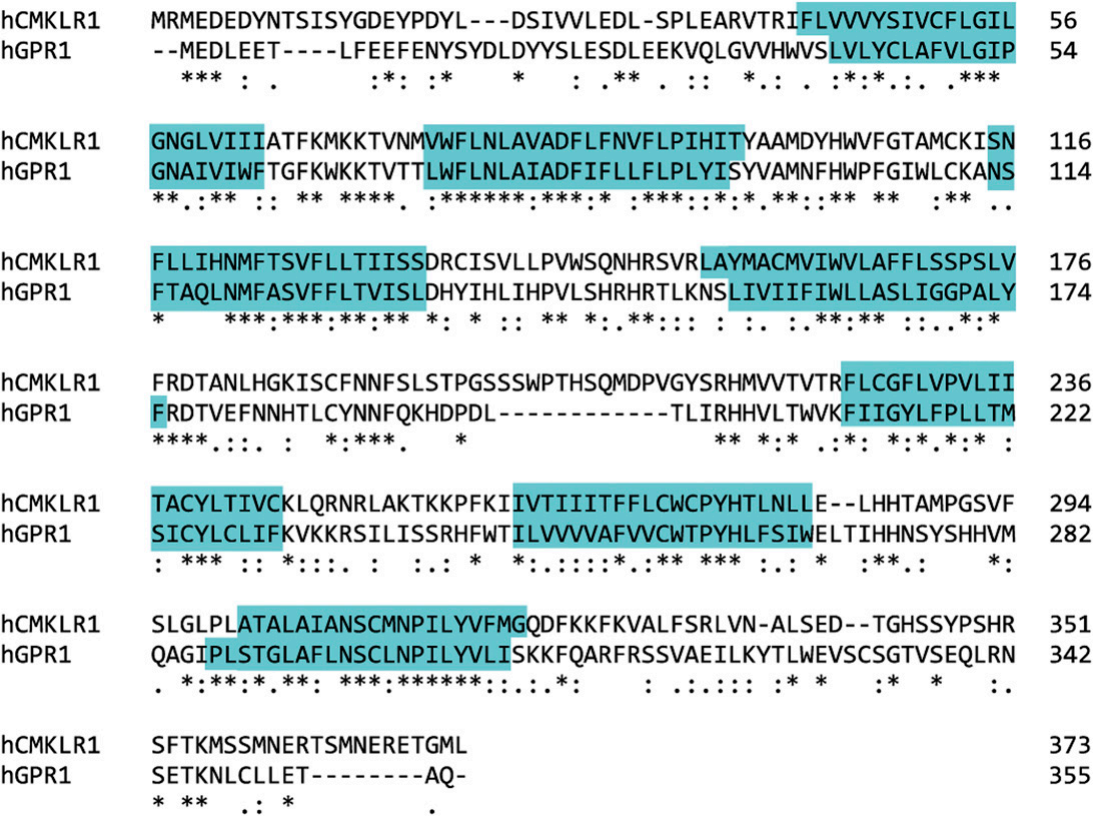
Clustal Omega (http://www.uniprot.org/align) sequence alignment of the human CMKLR1 and GPR1. Identical amino acids in all species, “*”; conserved amino acid substitution, “:”; and semiconserved amino acid substitution, “.” Blue boxes highlight the transmembrane domains.

GPR1 was originally cloned by Marchese et al. (1994b) from a cDNA library of the human hippocampus, and the deduced amino acid sequence revealed the expected seven-transmembrane domains (Fig. 5). The *GPR1* and *CMKLR1* genes share a common ancestor (Vassilatis et al., 2003) and have a sequence identity of 37% (Fig. 6). The similarities between GPR1 and CMKLR1 both at the amino acid and gene levels are shown to be present across various species from lower to higher vertebrate (fish to human). Porcine GPR1, like human GPR1 and porcine CMKLR1, is intronless in the coding region, and computational analysis suggests almost identical predicted three-dimensional structures for porcine GPR1 and CMKLR1 (Huang et al., 2010). Based on the similarities between the two receptors, Barnea et al. (2008) were the first to identify chemerin as a ligand for GPR1. They reported that radiolabeled C9 bound to GPR1 with a subnanomolar affinity, and both human chemerin21–157 and C13 activated the receptor in a *β*-arrestin recruitment assay with a similar nanomolar potency. This pairing has been independently confirmed by three different groups (Southern et al., 2013; Rourke et al., 2014; Kennedy et al., 2016). GPR1 has been suggested to be a functional chemerin receptor in vivo; characterization of the *GPR1* knockout mouse model revealed that GPR1 acts to modify glucose homeostasis during obesity, in agreement with known functions of chemerin (Rourke et al., 2014). In light of this, and consistent with International Union of Pharmacology convention, we recommend that GPR1 is officially paired with ligand chemerin and renamed accordingly. As CMKLR1 is chemerin receptor 1, GPR1 should be designated chemerin receptor 2 for ligand chemerin (Table 4).

**TABLE 4:** Classification of chemerin receptor 2

Receptor Structure, Pharmacology, and Distribution	Receptor Amino Acid Sequences, Pharmacological Parameters, Tissue Distribution	References
Previous names	GPR1	
Structural information	7TM	
Humans	355 aa (UniProt P46091) chr. 2q33.3 (Entrez 2825)	
Rats	353 aa (UniProt P46090) chr. 9q31 (Entrez 25457)	
Mice	353 aa (UniProt Q8K087) chr. 1C2 (Entrez 241070)	
Functional assays	CHO cells transfected with GPR1	Barnea et al., 2008; Kennedy et al., 2016
Endogenous agonists	Human chemerin(21–157) (pEC_50_∼9)	Barnea et al., 2008; Southern et al., 2013; Kennedy et al., 2016
Agonists	C9 [chemerin(149–157)] (pEC_50_ = 8.65 ± 0.14)	Kennedy et al., 2016
	C13 [chemerin(145–157)] (pEC_50_ = 9.05 ± 0.09)	Kennedy et al., 2016
	C20 [chemerin(138–157)]	Li et al., 2014a
Selective antagonist	None	
Radioligands	[^125^I]-C9 (K_D_ = 5.3 nM) (pIC_50_ = 9.3)	Barnea et al., 2008; Kennedy et al., 2016
	Human [^125^I]-chemerin(21–157) (K_D_ = 0.21 nM)	De Henau et al., 2016
Transduction mechanisms	Coupled to G_i/o_ proteins: predicted	Rourke et al., 2015
		
Receptor distribution		
Humans	Northern blot analysis identified the *GPR1* gene in the hippocampus	Marchese et al., 1994b
	RT-PCR showed highest expression of GPR1 mRNA in the adrenal cortex, cardiomyocytes, and superior cervical ganglion	Wu et al., 2009
	Immunohistochemistry confirmed GPR1 expression in smooth muscle cells of the vasculature	Karagiannis et al., 2013; Kennedy et al., 2016
Mice	RT-PCR identifed highest GPR1 mRNA expression in white adipose tissue (predominately the stromal vascular fraction), with high levels also detected in skin, brown adipose tissue, skeletal muscle, the brain (particularly the hypothalamus), bladder, esophagus, and ovaries	Regard et al., 2008; Takahashi et al., 2011; Rourke et al., 2014; Yang et al., 2016
Rats	RT-PCR detected GPR1 mRNA in the male reproductive system, with high levels in the testis	Li et al., 2014b
Tissue function	HIV/SIV coreceptor; lipid metabolism	Edinger et al., 1997; Shimizu et al., 2009; Rourke et al., 2014

aa, Amino acid; chr., chromosome; RT-PCR, reverse-transcription polymerase chain reaction; SIV, simian immunodeficiency virus.

**Fig. 7.**
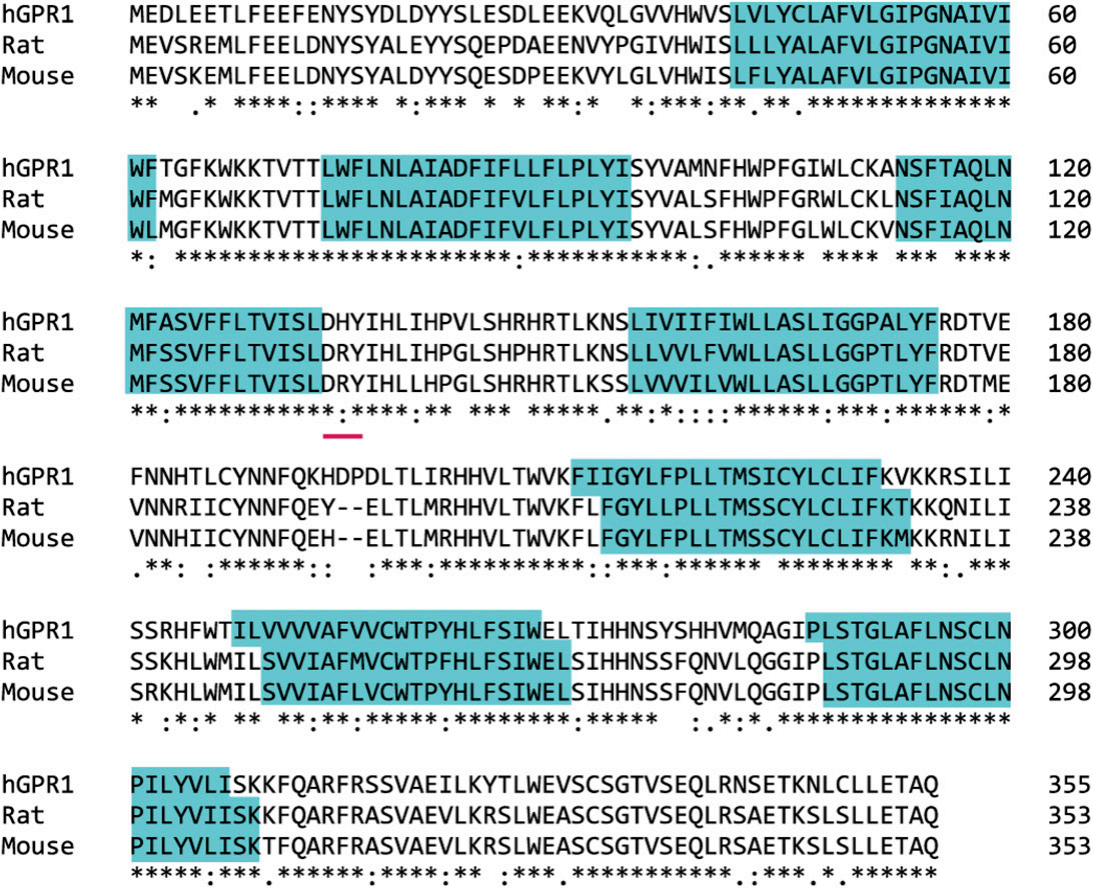
Clustal Omega (http://www.uniprot.org/align) sequence alignment of the human, rat, and mouse chemerin receptor 2. Identical amino acids in all species, “*”; conserved amino acid substitution, “:”; and semiconserved amino acid substitution, “.” Blue boxes highlight the transmembrane domains; the pink line identifies the G protein–binding motif; and the box highlights the differing sequence in humans.

Chemerin receptor 2 is a class A GPCR, specifically the A4 subfamily, but as yet it is largely unknown what G protein pathway it activates. One study showed that chemerin modestly induced calcium release (Barnea et al., 2008). A further study found that chemerin stimulates RhoA signaling, which was sensitive to pertussis toxin (PTX), a highly selective G*α*_i/o_ inhibitor (Rourke et al., 2015), suggesting that, similar to CMKLR1, this receptor could be coupled to G_i/o_ (see *Section VIII. B. Signaling Pathways Activated by Chemerin Receptor 2*). Human chemerin receptor 2 shares 79% and 80% sequence identity with rat and mouse chemerin receptor 2, respectively (Fig. 7).

## IV. Distribution

A very limited number of studies have investigated the expression of both CMKLR1 and GPR1 in the same tissue. To begin to understand whether these chemerin receptors are expressed differentially or are present within the same tissues, future studies should identify both receptors and consider not only the proximity to ligand chemerin but whether the receptors colocalize. In addition, an important limitation in the studies of chemerin expression is that they do not differentiate between active and inactive chemerin isoforms (discussed further in *Section VI. A. Chemerin Receptor 1 Agonists*). Subsequent studies should endeavor to state which isoform of chemerin is identified using specific antibodies.

### A. Chemerin Receptor 1

#### 1. Humans

In humans, reverse-transcriptase quantitative polymerase chain reaction has identified that the *CMKLR1* gene is predominately expressed in dendritic cells, monocytes, and macrophages (Wittamer et al., 2003; Herová et al., 2015) with high expression also seen in adipose tissue, spleen, lymph nodes, lung, skin, adipocytes, vascular smooth muscle cells, endothelial cells, and natural killer cells (Wittamer et al., 2003; Goralski et al., 2007: Parolini et al., 2007; Kaur et al., 2010; Kostopoulos et al., 2014; Banas et al., 2015; Kennedy et al., 2016). Further analysis of CMKLR1 mRNA in the skin showed it is located predominately to the dermal layer (Banas et al., 2015). CMKLR1 mRNA was abundantly expressed in adipose tissues (Roh et al., 2007; Catalán et al., 2013). Immunostaining followed by fluorescence-activated cell sorter analysis confirmed that CMKLR1 is expressed on the cell surface of dendritic cells, monocytes, and macrophages (Wittamer et al., 2003; Vermi et al., 2005; Zabel et al., 2005b; Herová et al., 2015). In agreement with the mRNA expression pattern, immunohistochemistry localized CMKLR1 expression to smooth muscle cells and the surrounding adipose tissue of human vessels (aorta, saphenous vein, coronary artery, mammary arteries, and resistance vessels) (Kostopoulos et al., 2014; Kennedy et al., 2016), lung bronchioles (Kennedy et al., 2016), lymph nodes (Vermi et al., 2005), as well as the testis (Li et al., 2014b). Western blot detected high CMKLR1 protein from endothelial cells (Kaur et al., 2010).

#### 2. Mice

In mice, CMKLR1 mRNA levels were highest in white adipose tissue and lung, with lower levels of expression seen in heart, placenta, kidney, spleen, brain, liver, testis, skin, ovary, mesenteric lymph nodes, colon, and thymus (Goralski et al., 2007; Luangsay et al., 2009; Rourke et al., 2014). CMKLR1 mRNA was significantly higher in white adipose tissue compared with brown adipose tissue (Goralski et al., 2007). Although expression was abundant in both the adipocytes and the stromal vascular fraction of white adipose tissue (Rourke et al., 2014), there were twofold higher CMKLR1 mRNA levels in adipocytes and protein expression was confirmed using immunohistochemistry (Goralski et al., 2007). Further clarification of the expression in skin shows that mRNA levels are higher in the dermal layer (Banas et al., 2015). CMKLR1 protein has also been detected in the *β*-cells in the islets of the mouse pancreas (Takahashi et al., 2011). CMKLR1 mRNA and protein were detected in murine cardiomyocytes (Rodríguez-Penas et al., 2015). Monoclonal antibody staining of ChemR23 showed high expression in immature plasmacytoid dendritic cells and at lower levels in myeloid dendritic cells, macrophages, and natural killer cells (Zabel et al., 2006; Luangsay et al., 2009).

#### 3. Rats

In rats, CMKLR1 is expressed in the reproductive system. High levels of CMKLR1 mRNA were detected in the testis (Li et al., 2014b) and ovaries (Wang et al., 2012). This was consistent with immunostaining of CMKLR1 expression in Leydig cells (Li et al., 2014b). In addition, CMKLR1 is expressed in the rat vasculature. Western blot analysis showed that CMKLR1 was present in endothelial cells (Zhao et al., 2013), cardiomyocytes (Zhang et al., 2014), as well as aorta and mesenteric arteries, and immunostaining confirmed that expression was in the smooth muscle media layer and the endothelium (Watts et al., 2013).

#### 4. Other Species

In cows, CMKLR1 mRNA has been detected in adipose tissue, liver, and mammary gland (Suzuki et al., 2015), as well as various ovarian cells, including granulosa and theca cells, corpus luteum, and oocytes (Reverchon et al., 2014). In ovarian cells from turkeys, CMKLR1 mRNA was mostly present in theca cells rather than granulosa cells (Diot et al., 2015). In pigs, CMKLR1 mRNA was present in white adipose tissue and spleen, with low levels detected in stomach and lung (Huang et al., 2010).

### B. Chemerin Receptor 2

#### 1. Humans

In humans, GPR1 was discovered when Northern blot analysis identified GPR1 mRNA in the hippocampus (Marchese et al., 1994b). The same study reported that GPR1 mRNA was not present in other brain tissues, including the cerebellum, frontal cortex, and thalamus, or other peripheral tissues such as macrophages and monocytes. GPR1 mRNA is also present in primary brain-derived fibroblast-like cell lines such as BT-3 and BT-20/N (Shimizu et al., 1999), mesangial cells (Tokizawa et al., 2000), U87 glioblastoma cells, and alveolar macrophages (Farzan et al., 1997; Yamaguchi et al., 2011). According to the BioGPS database, GPR1 mRNA is ubiquitously expressed in humans, with the highest expression seen in the adrenal cortex, cardiomyocytes, and superior cervical ganglion (Wu et al., 2009). Reverse-transcriptase quantitative polymerase chain reaction and immunohistochemistry identified GPR1 mRNA and protein expression in smooth muscle cells of human vessels (aorta, saphenous vein, coronary artery, mammary arteries, and resistance vessels), as well as lung bronchioles and kidney tubules (Karagiannis et al., 2013; Kennedy et al., 2016). GPR1 mRNA has also been identified in skin (Banas et al., 2015).

#### 2. Mice

Similar to CMKLR1, in mice, the highest GPR1 mRNA levels were found in white adipose tissue (Takahashi et al., 2011); however, GPR1 had a more ubiquitous expression, with high levels also seen in skin, brown adipose tissue, skeletal muscle, the brain (particularly the hypothalamus), bladder, and esophagus, and lower levels detected in heart, lung, liver, kidney, spleen, and thymus (Regard et al., 2008; Rourke et al., 2014). In contrast to CMKLR1, GPR1 was predominately expressed in the stromal vascular fraction of white adipose tissue, not the adipocytes (Rourke et al., 2014). GPR1 mRNA was also detected in the ovaries, more precisely with high protein expression in the oocytes of developing follicles, and in the thecal cells, granulosa cells, luteal cells, and interstitial cells of the stroma (Yang et al., 2016).

#### 3. Rats

To date, only one study has characterized the expression pattern of GPR1 in rats, specifically in the male reproductive system. GPR1 mRNA was most highly expressed in the testis, with moderate expression in prostate and epididymis fat (Li et al., 2014b). As a control tissue, the authors also report very little expression of GPR1 mRNA in the liver.

#### 4. Other Species

Like CMKLR1 expression, GPR1 mRNA was detected in adipose tissue, liver, and mammary gland, and ovarian cells in cows (Reverchon et al., 2014; Suzuki et al., 2015) and ovarian cells in turkeys (Diot et al., 2015). In pigs, GPR1 was expressed in kidney, white adipose tissue, spleen, and liver (Huang et al., 2010).

### C. Chemerin

#### 1. Humans

In humans, a RNA array discovered that chemerin mRNA was widely expressed in many tissues. The highest expression was seen in the liver, pancreas, adrenal gland, and skin, but expression was also detected in lymph nodes, heart, and ileum (Zabel et al., 2005b). Another study identified that high levels of chemerin mRNA were present in the liver, lung, pituitary gland, and ovary (Wittamer et al., 2003). This is consistent with the BioGPS database, which identified the most chemerin mRNA in liver, adrenal cortex, and lung (Wu et al., 2009). Further studies into the precise location of chemerin mRNA in the skin revealed that, in contrast to its receptors, chemerin is predominately expressed in the epidermis (Banas et al., 2015). Northern blot analysis confirmed that chemerin mRNA was highly expressed in liver and pancreas (Takahashi et al., 2011). Immunohistochemistry localized chemerin to the cardiovascular system, including the pericoronary and periaortic adipose tissue, specifically to adipocytes and stromal vascular cells (Kostopoulos et al., 2014). In addition, chemerin was detected in vascular smooth muscle cells and endothelial cells of human vessels (aorta, saphenous vein, coronary artery, mammary arteries, and resistance vessels), endocardial endothelial cells, epithelial cells of the lung bronchioles, and tubules and endothelial cells in the kidney (Kennedy et al., 2016).

#### 2. Mice

In mice, similar to that found in humans, chemerin mRNA was detected in a wide range of different tissues. The highest expression was seen in liver, skin, white adipose tissue, and placenta (Goralski et al., 2007; Banas et al., 2015), but chemerin mRNA was also present in ovary, skin, lung, liver, ilieum, colon, testis, thymus, spleen, mesenteric lymph nodes, and Peyer’s patches (Luangsay et al., 2009; Yang et al., 2016). Chemerin mRNA levels were significantly higher in white adipose tissue compared with brown, and they were more abundant in adipocytes than in the stromal vascular fraction of white adipose tissue, a similar expression pattern to its receptor CMKLR1 (Goralski et al., 2007). Also, like CMKLR1, chemerin protein was localized to *β*-cells in islets of the pancreas (Takahashi et al., 2011).

#### 3. Rats

In rats, chemerin mRNA is predominately expressed in the liver and adipose tissue (Watts et al., 2013; Li et al., 2014b). Immunohistochemistry confirmed expression of chemerin in the perivascular adipose tissue surrounding aorta and mesenteric arteries (Watts et al., 2013). Chemerin mRNA has also been detected in rat ovaries (Wang et al., 2012).

#### 4. Other Species

Similar to other species, high levels of chemerin mRNA were seen in adipose tissue and liver of cows (Suzuki et al., 2015) and pigs (Huang et al., 2010) as well as liver of turkeys (Diot et al., 2015). The expression pattern of chemerin mRNA in ovarian cells from cows and turkeys is consistent with that of its receptors (Reverchon et al., 2014; Diot et al., 2015).

## V. Radiolabeled Ligands

Two independent studies have carried out saturation-binding analysis in cells artificially expressing either human CMKLR1 or human GPR1 to determine the dissociation constant, K_D_ values of [^125^I]Tyr^149^-C9 at each receptor. Barnea et al. (2008) report K_D_ values for CMKLR1 and GPR1 as 4.9 and 5.3 nM, respectively, and show that both human chemerin21–157 (IC_50_ = 0.15 nM, CMKLR1 and 0.23 nM, GPR1) and unlabeled C13 (IC_50_ = 1.9 nM, CMKLR1 and 2.3 nM, GPR1) compete for all the binding at both receptors. Kennedy et al. (2016) report K_D_ values of 0.3 and 0.9 nM for CMKLR1 and GPR1, respectively, and found that unlabeled C9 competed for all the binding at both receptors (pK_i_ = 9.21 ± 0.14, CMKLR1 and 8.81 ± 0.17, GPR1). Subsequent studies in human cardiovascular tissues showed ^125^I-C9 bound specifically, saturably, and reversibly to a single site in human saphenous vein, identified as CMKLR1, with a K_D_ = 0.53 ± 0.31 nM and B_max_ = 0.05 ± 0.007 fmol/mg and a hill slope of 1 (Kennedy et al., 2016).

Further characterization of human CMKLR1 has been carried out with other radiolabeled ligands in transfected cells. Derived from human chemerin146–157, in which Tyr^149^ has been replaced by a phenylalanine and an N-terminal tyrosine added, the peptide YHSFFFPGQFAFS was radioiodinated on tyrosine residue, [^125^I]Tyr-[Phe^149^]-chemerin146–157 (Wittamer et al., 2003). [^125^I]Tyr-[Phe^149^]-chemerin146–157 bound to cells transfected with human CMKLR1 with a K_D_ = 22 nM calculated using a single-site model. Chemerin21–157, chemerin139–157, and C9 (pIC_50_ = 8.18 ± 0.27; 7.70 ± 0.07; and 8.26 ± 0.09, respectively) all competed for radiolabeled binding with a similar affinity (Wittamer et al., 2004).

Although the shorter fragments of chemerin are useful tool compounds, further study into the binding of full-length human chemerin to human CMKLR1 and GPR1 is required to understand how the endogenous ligand binds to its receptors. Although there are the obvious limitations of radiolabeling a 143-amino-acid protein, two groups have shown that larger fragments of chemerin can be radiolabeled in their studies on mouse CMKLR1. Radiolabeled human chemerin21–148 bound to native CMKLR1 receptors on wild-type (WT) mouse macrophages with a calculated K_D_ = 1.2 nM, and binding to macrophages from *CMKLR1*-deficient mice was significantly reduced (Bondue et al., 2012). Similarly, Zabel et al. (2008) show binding of this radiolabel to mouse CMKLR1 with an EC_50_ = 3.1 nM. Most recently, De Henau et al. (2016) have importantly shown that human [^125^I]chemerin(21–157) binds to Chinese hamster ovary (CHO)-K1 cells transfected with CMKLR1 and GPR1 with K_D_ of 0.88 and 0.21 nM, respectively.

Subsequent studies should investigate binding to the receptor with the endogenous ligand from the same species. The structure of chemerin is described as a mirror-image, reverse chemokine (Zabel et al., 2006) with an N-terminal core region and a flexible C-terminal region that needs to be cleaved for receptor activation. It would therefore be insightful to understand how the different regions bind to the receptors and whether this in turn alters the downstream signaling pathways that are activated. Chemokines bind to their receptors in a two-site model (Clark-Lewis et al., 1995), and chemerin binding could be similar. It has emerged that the shorter C-terminal fragments of chemerin are biased toward the G protein pathway compared with *β*-arrestin (see *Section VIII. A. Signaling Pathways Activated by Chemerin Receptor 1*); therefore, the N-terminal core region of chemerin could be required for activation of the latter pathway.

In addition, [^3^H]-resolvin E1 (RvE1) binds to a single site on CMKLR1-transfected cells with a high affinity (K_D_ = 11.3 ± 5.4 nM, B_max_ = 4200 ± 1050 binding sites per cell), which is blocked by 10 *µ*M chemerin peptide (Tyr-[Phe^149^]-chemerin146–157), suggesting that they have the same recognition site within the receptor (Arita et al., 2005).

## VI. Agonists

### A. Chemerin Receptor 1 Agonists

The endogenous agonist of CMKLR1 is the chemoattractant chemerin derived from the *RARRES2* gene. Wittamer et al. (2003) isolated an active fraction of human ascitic fluids and found that, after tryptic digest and mass spectrometry, eight peptide products were predicted to originate from the *RARRES2* gene. The C-terminal peptide was not tryptic, suggesting that the active isoform, lacking six amino acids, was a result of proteolytic cleavage of a precursor. Chemerin21–157 bound to and was functionally active at the CMKLR1 receptor with a low nanomolar potency (EC_50_ = 4.5 ± 0.7 nM), whereas precursor, prochemerin, was 50–100× less potent (EC_50_ = 393 ± 116) (Wittamer et al., 2003). Meder et al. (2003) similarly identified that a product of the RARRES2 gene, modified at the N and C terminus, activated CMKLR1; however, they found that the circulating active form was chemerin21–154 (no EC_50_ value given). Human serum had abundant chemoattractant activity at CMKLR1 due to circulating chemerin21–155 (no EC_50_ value given), an eight C-terminal amino acid truncation of prochemerin (Zabel et al., 2005b). The activity of chemerin in serum was lost on addition of protease inhibitors, confirming that proteolytic processing of the C terminus is required to convert prochemerin into the bioactive form, and this occurs post-translationally by various serine (Zabel et al., 2005a) and cysteine proteases (Kulig et al., 2011). It is not well characterized what the active fragment of chemerin is in humans, or whether different lengths are found in different environments and have different functions. This is a key area of research that needs to be understood in the field of chemerin and its receptors. Like in the localization studies, many studies comparing plasma levels of chemerin with disease measure chemerin immunoreactivity without differentiating whether it is prochemerin, active chemerin, or degradation products. Future experiments should consider that prochemerin is not biologically active, and therefore measurements of chemerin levels should precisely identify cleavage of this precursor. This in turn will give us important knowledge regarding chemerin as an agonist, and the active fragments can then be characterized pharmacologically.

The studies by Meder et al. (2003) and Zabel et al. (2005b) did not characterize the pharmacology of chemerin21–154 and chemerin21–155 at the human CMKLR1 receptor. Their functional findings are in contrast to later work by Wittamer et al. (2004), who carried out extensive in vitro studies to identify the residues of chemerin necessary for activation and function at human CMKLR1. Peptides of different lengths were synthesized, and their activities tested against human CMKLR1-transfected cells in a calcium assay. Chemerin139–157 (EC_50_ = 16.7 ± 3.2 nM) retained most of the activity of chemerin21–157, and, as expected, the prochemerin equivalents, chemerin139–163 and chemerin158–163, lost all biologic activity. Interestingly, removal of one, two, or three amino acids, chemerin139–156 (EC_50_ = 97 ± 13 nM), chemerin139–155, and chemerin139–154, or addition of one amino acid, chemerin139–158, all led to a significant drop in potency. This confirms that precise cleavage at the C terminus to the serine residue at position 157 is critical for activity. In agreement, the work of Li et al. (2014a) reports C20, chemerin138–157 (competition binding IC_50_ = 1.6 nM, functional response from 1 nM), as a potent agonist at CMKLR1. C20 corresponds to an evolutionary conserved region of the mature protein flanked by potential cleavage sites. Further C-terminal peptides from the human sequence were synthesized, by Wittamer et al. (2004), to identify the shortest peptide with the highest potency. Truncating the N terminus to give C13 (pD_2_ = 7.85 ± 0.01) or C9 (pD_2_ = 8.15 ± 0.02) retained similar activity to human chemerin21–157; however, further removal of the N-terminal tyrosine to the eight-amino-acid peptide, chemerin150–157, resulted in a significant drop in potency. Alanine-screening mutagenesis identified the aromatic residues Tyr^149^, Phe^150^, Phe^154^, and Phe^156^, as well as Gly^152^, as important residues that likely contribute to direct interaction between chemerin and CMKLR1 (Wittamer et al., 2004).

Similarly, to investigate the binding and function of chemerin at mouse CMKLR1, Cash et al. (2008) synthesized different fragments derived from the C terminus of mouse chemerin. They identified that C15 retained most of the activity of mouse chemerin17–156 in a macrophage chemotaxis assay (no EC_50_ values given); however, mouse chemerin144–154, chemerin144–156, and chemerin138–156 were significantly less effective or had no effect in inhibiting induced cytokine production. The authors have since shown that C15 enhanced macrophage phagocytosis, leading to improved microbial particle clearance and apoptotic neutrophil ingestion (Cash et al., 2010), and inhibited intravascular inflammatory events to accelerate wound closure and reduce scarring (Cash et al., 2014). However, over the course of these studies, the pharmacology of this peptide has not been characterized, and these findings are in contrast to others who report no activity in cells transfected with CMKLR1. Shimamura et al. (2009) found that amino acids Phe^155^ and Ser^156^ in mouse chemerin were essential for activity at the mouse CMKLR1 receptor, and that the equivalent mouse peptide to human C9, mouse chemerin148–156, activates the mouse receptor with comparable potency to mouse chemerin17–156. Rourke et al. (2015) also report that C15 did not activate the serum-response factor luciferase signaling assay or *β*-arrestin recruitment via either mouse or human CMKLR1. C15 is therefore a controversial agonist of the CMKLR1 receptor; many academic and industry groups are unable to reproduce the findings that this is an active compound. As they have not yet been performed, must-do experiments to clarify the role of this peptide include radioligand saturation and competition-binding experiments and pharmacological profiling in different signaling assays, with negative results as valuable as positive.

As discussed in *Section VIII. A. Signaling Pathways Activated by Chemerin Receptor 1*, biased signaling has been reported at CMKLR1 (Kennedy et al., 2016). This selective activation of a downstream pathway over another by different agonists could be an explanation for the at times confusing and contradictory results seen for chemerin agonists at CMKLR1. Human chemerin21–155, human chemerin21–154, and C15, reported to be functionally active, may not activate the specific downstream signaling pathways tested by others, and thus appear inactive in those studies. To understand this, the full pharmacology of chemerin agonists in binding and multiple downstream signaling pathways needs to be carried out. CellKey or EPIC dynamic mass redistribution assays that monitor activation of all downstream signaling pathways should be used to confirm activation of the receptor by these controversial agonists.

RvE1, an oxygenated product of the essential fatty acid eicosapentaenoic acid, is a potent anti-inflammatory and proresolving mediator and has been shown to activate CMKLR1. Arita et al. (2005) found that in CMKLR1-transfected HEK293 cells, RvE1 caused a concentration-dependent inhibition of tumor necrosis factor (TNF)-*α*–induced nuclear factor-*κ*B activation with an EC_50_ of 1.0 nM. Further studies by the same laboratory confirmed that RvE1 binds to CMKLR1-transfected CHO cells in a concentration-dependent manner (Ohira et al., 2010) and stimulates *β*-arrestin recruitment (EC_50_ = 13 pM) (Krishnamoorthy et al., 2010) and adenosine diphosphate activation of human platelets in a CMKLR1-dependent manner (Fredman et al., 2010). One independent study has identified the same pairing, showing that RvE1-induced proliferation of fibroblasts was inhibited by CMKLR1 small interfering RNA (Qu et al., 2012), but others were unable to reproduce these findings (Luangsay et al., 2009; Davenport et al., 2013). Bondue et al. (2011b) also suggest that there is uncertainty because a later study by Arita et al. (2007) identified a structurally similar receptor, leukotriene B4 receptor BLT1, that mediates RvE1 activities. Although a previous review is convinced (Bäck et al., 2014), to date the pairing of RvE1 and CMKLR1 has not been confirmed, and further studies are required to clarify this area.

One study (Peng et al., 2015) has shown that the amyloid-*β* peptide (A*β*42) conjugated to fluorescein isothiocyanate binds to rat basophilic leukemia cells stably transfected with CMKLR1 with K_D_ = 0.8 ± 0.2 *µ*M. The authors report functional activity of A*β*42 (maximum at 2 *µ*M), similar to C9 in induction of chemotaxis of primary microglia cells. However, further studies showed that A*β*42 and C9 stimulated different pathways in this cell line, A*β*42 does not induce Ca^2+^ release, but induced chemotaxis, whereas C9 induced Ca^2+^ and chemotaxis. No other groups have reported actions of A*β*42 on CMKLR1, and so confirmation of this pairing is required.

### B. Chemerin Receptor 2 Agonists

Recombinant human chemerin and C-terminal peptides, C9 and C13, are the only known agonists of GPR1. *β*-arrestin recruitment studies on cells transfected with human GPR1 have given insight into its potential endogenous agonists. Human chemerin21–157 has been reported to be a potent agonist at human GPR1 with a subnanomolar potency (pD_2_ = 9.6, Barnea et al., 2008; pD_2_ = 8.8, Southern et al., 2013; pD_2_ = 9.1, Kennedy et al., 2016), with one group reporting a lower potency of pD_2_ = 7.7 (Rourke et al., 2014). C9 and C13 activate GPR1 with a similar potency to chemerin, pD_2_ = 8.09 ± 0.16, 8.65 ± 0.14, and 9.05 ± 0.09, respectively; however, they appear to be partial agonists compared with chemerin, with lower maximum responses, E_max_ = 71% ± 5%, 97% ± 3%, and 134% ± 6% response of C13, respectively (Kennedy et al., 2016). In addition, human chemerin21–157 and mouse chemerin17–156 have been shown to activate gene expression via both human and mouse GPR1 in vitro (Rourke et al., 2015). The next step is to confirm that endogenous chemerin, present in humans, binds and activates GPR1, and, as for CMKLR1, it will then be vital to begin to understand what lengths of chemerin are found endogenously and whether different lengths have different functions via this receptor.

## VII. Antagonists

C-terminal processing of prochemerin has resulted in a number of agonists with different affinities and potencies. One hypothesis that has emerged is that the different isoforms compete at the chemerin receptors to exert their actions through different signaling pathways. One group identified that chemerin21–155 had no agonistic activity at CMKLR1 but inhibited chemerin21–157–induced Ca^2+^ flux by 50% (Yamaguchi et al., 2011), suggesting that it is acting as antagonist. Similarly, Peng et al. (2015) report that C15 blocked the response A*β*42 at CMKLR1. This complicates further the nature of chemerin processing and signaling, but could also offer a reason why C15 appears to have anti-inflammatory effects (Cash et al., 2008) in contrast to proinflammatory actions of other isoforms (Wittamer et al., 2003). This idea can be easily investigated pharmacologically, using cells transfected with CMKLR1 or GPR1 and different signaling readouts; known chemerin agonists can be incubated as antagonists first before addition of a second chemerin agonist.

A small-molecule antagonist, CCX832, designed and synthesized by Chemocentryx (Mountain View, CA), is reported to be a CMKLR1 antagonist. It has a nanomolar IC_50_ for the receptor in humans (2.4 nM), rats (2 nM), and mice (5 nM) calculated through radiolabeled binding assays, and similar affinities were found in functional calcium mobilization and chemotaxis experiments (Watts et al., 2013). Further characterization of the compound was carried out on CHO cells transfected with either human CMKLR1 or GPR1 to explicitly confirm its selectivity both in binding and functional assays. Kennedy et al. (2016) showed that CCX832 competed for radiolabeled C9 binding to CMKLR1 (pK_i_ = 9.16 ± 0.42), but not GPR1, and that CCX832 blocked C9-mediated *β*-arrestin recruitment at CMKLR1 (pA_2_ = 8.32 ± 0.04), but had no effect on the C9 response at GPR1. Further confirmation of the compound selectivity has been highlighted by using silencing RNA. In mesenchymal stromal cells, chemerin significantly increased cell migration, and this effect was blocked by CCX832, mimicking the effect of CMKLR1 knockdown cells, whereas GPR1 knockdown cells had no effect on the chemerin response (Kumar et al., 2014). CCX832 has been demonstrated to block chemerin-induced vasoconstriction in rat aorta (Watts et al., 2013) and human vessels (Kennedy et al., 2016) in vitro, inhibit chemerin-induced increase in mean arterial blood pressure in rats in vivo (Kennedy et al., 2016), and affect cell migration in the development of cancers (Kumar et al., 2014, 2016). CCX832 is therefore a highly selective CMKLR1 antagonist and can be used as a definitive means to differentiate the response of chemerin at chemerin receptor 1.

One further small-molecule 2-(anaphthoyl) ethyltrimethylammonium iodide (*α*-NETA; Fig. 1D), originally identified as a choline acetyltransferase inhibitor, has also been reported as a CMKLR1 antagonist (Graham et al., 2014). Measuring *β*-arrestin recruitment, using CHO cells transfected with human CMKLR1, *α*-NETA had an IC_50_ of 0.38 *µ*M. In experimental autoimmune encephalomyelitis, a model for human multiple sclerosis, *α*-NETA significantly delayed the onset of the disease in agreement with that seen in *CMKLR1*-deficient mice. The authors note the limitations of selectivity with this compound, specifically that *α*-NETA has an IC_50_ of 3.4 *µ*M to GPR1-transfected cells, only 10-fold less potent than CMKLR1.

Most recently, nanobodies (CA4910 and CA5183) targeted toward the CMKLR1 receptor have been developed (Peyrassol et al., 2016). The authors report that these nanobodies bind to the same binding site as chemerin on CMKLR1 and antagonize chemerin-mediated intracellular calcium release and chemotaxis of human monocyte-derived dendritic cells.

The existence of two chemerin receptors necessitates that for each function of chemerin the target needs to be explicitly characterized through imaging of receptor distribution and pharmacologically. However, a lack of available selective antagonists for these receptors is a current struggle in the field. At this stage, it is only possible to decisively state functionality through CMKLR1 using selective compound CCX832, which is not commercially available. There is not yet an antagonist for GPR1, and this needs to be developed to identify the function of GPR1 in humans. Further pharmacological studies are necessary to identify differences in amino acid residues activated in the GPR1 receptor by chemerin compared with CMKLR1 that could be exploited to design and synthesize selective antagonists. Based on the receptor sequences, CMKLR1 (Fig. 2) has a larger extracellular loop 2 than GPR1 (Fig. 5). It is not yet known what this means pharmacologically, but this could be useful in the development of selective compounds. An alternative would be to carry out a high-throughput screen using expressed GPR1 receptors, but this may require further evidence that blocking GPR1 would represent a new therapeutic target to justify investment.

## VIII. Receptor Signaling

### A. Signaling Pathways Activated by Chemerin Receptor 1

Current knowledge of the signaling pathways downstream of CMKLR1 has predominately come from assays in cell lines and demonstrates that this receptor couples to the G*α*_i/o_ signaling pathway, causing release of intracellular Ca^2+^ (Wittamer et al., 2003; Kaneko et al., 2011) and inhibition of cAMP accumulation (Kennedy et al., 2016). A study using bioluminescence resonance energy transfer–based biosensors confirmed that chemerin21–157 and C9 activated three G*α*_i_ subtypes (G*α*_i1_, G*α*_i2_, and G*α*_i3_) and the two G*α*_o_ isoforms (G*α*_oa_ and G*α*_ob_) with a comparable potency to the binding affinity (De Henau et al., 2016). In addition, a number of studies have reported downstream signaling through other pathways, including phosphorylation of extracellular signal-regulated kinases 1/2 and MAPK (Wittamer et al., 2003; Goralski et al., 2007; Sell et al., 2009; Hart and Greaves, 2010; Kaneko et al., 2011; Lobato et al., 2012) and activation of RhoA (Rourke et al., 2015), all of which are sensitive to PTX, and *β*-arrestin recruitment (De Henau et al., 2016; Kennedy et al., 2016). Experiments identifying which residues in the human receptor are involved in downstream signaling have not yet been carried out. At the rat receptor, it has been shown that Ser^343^ is necessary for phosphorylation by GPCR kinase and Ser^347^ by phosphokinase C (Zhou et al., 2014).

Most recently, it has been discovered that chemerin agonists can selectively signal through different pathways via CMKLR1. Intriguingly, C9 and C13 were more potent (pD_2_ = 9.39 ± 0.09 and 9.12 ± 0.12, respectively) than human chemerin21–157 (pD_2_ = 8.45 ± 0.10) in G protein–dependent cAMP inhibition assays, but less potent (pD_2_ = 7.09 ± 0.06, and 7.15 ± 0.04, respectively) at inducing *β*-arrestin recruitment compared with chemerin21–157 (pD_2_ = 9.37 ± 0.05) (Kennedy et al., 2016). C9 and C13 preferentially activated cAMP inhibition with a bias factor of ∼5000. This could suggest a trend for shorter C-terminal fragments of chemerin to be strongly biased toward activation of G protein pathway. Biased signaling was previously proposed at the CMKLR1 receptor. Peng et al. (2015) presented A*β*42 as a biased ligand due to its ability to promote chemotaxis and internalize CMKLR1, but, unlike other chemerin ligands, it had no effect on Ca^2+^ flux, and Rourke et al. (2015) hypothesized the possibility, although it was beyond the scope of their downstream signaling study. This finding suggests that endogenous chemerin fragments could have the potential to exhibit a spectrum of activities at different signaling pathways and could offer an alternative explanation for differing activities of chemerin agonists (see *Section VI. A. Chemerin Receptor 1 Agonists*).

It is possible that there is crosstalk between chemerin and other cytokines and their receptors. For example, it has been reported that CMKLR1 can form heterodimers with chemokine receptor CCR7 and CXCR4 (de Poorter et al., 2013) and that chemerin signaling synergises with CCL7 to produce significantly greater monocyte-derived dendritic cell migration than the additive response of the two ligands alone (Gouwy et al., 2014). Although this has not been reported specifically for CMKLR1 (as opposed to GPR1), it is likely in monocyte-derived dendritic cells that CMKLR1 is present. As seen for other cytokines, activation of a GPCR stimulates multiple coinciding signaling pathways that could synergise within the cell to give an amplified cellular response (Gouwy et al., 2005). In primary human hepatocytes, CMKLR1 was upregulated by the adipokine, adiponectin, with receptor expression significantly reduced in liver of adiponectin-deficient mice (Wanninger et al., 2012). In contrast, adiponectin downregulated CMKLR1 in bovine mammary epithelial cells (Suzuki et al., 2015). This suggests that other adipokines could influence CMKLR1 expression.

It has been implicated that CMKLR1 may signal through BMP4 to regulate estrogen and progesterone secretion in polycystic ovary syndrome (Tang et al., 2016), although this mechanism has not been fully delineated.

### B. Signaling Pathways Activated by Chemerin Receptor 2

The high-sequence identity of GPR1 with CMKLR1 has not only led to the pairing of this receptor with chemerin, but also allowed extrapolation of what signaling pathways the orphan receptor couples to. The G protein–coupling specificity of GPR1 was predicted using the receptor sequences from humans and mice and PRED-COUPLE 2.0 software. The results showed that both GPR1, in humans and mice, was predicted to couple to G*α*_q/11_ and G*α*_i/o_ (Rourke et al., 2015). In agreement with this prediction, the authors used cell-based assays with either human or mouse GPR1-transfected cells and found that chemerin activated the RhoA/RhoA kinase signaling at GPR1 and this was completely blocked by PTX. This suggests that, like CMKLR1, GPR1 is coupled to G*α*_i/o_. In addition, GPR1 was also found to modestly activate the MAPK/extracellular signal-regulated kinase pathway. At this early stage of discovery, much is still to be elucidated around GPR1 signaling, as yet no studies have identified specific residues involved in receptor signaling.

GPR1 has a mutation in G protein–binding motif DRY: a highly conserved motif at the end of transmembrane helix 3 involved in GTP/GDP exchange in G protein activation (Oldham and Hamm, 2008). A study by Rovati et al. (2007) shows that a nonconservative arginine mutation does not affect arrestin recruitment; however, it disrupts receptor function by reducing agonist-induced G protein activity. In human GPR1, the motif is mutated to DHY (Figs. 5 and 7), the histamine residue; although regarded as a conservative replacement of the arginine residue, it could affect G protein binding and therefore signaling at this receptor. Barnea et al. (2008) report that activation of GPR1 stimulated Ca^2+^ flux; although it was at low levels compared with that exerted by CMKLR1 and in CellKey experiments, human chemerin21–157, C9, and C13 were less potent at activating GPR1 than in *β*-arrestin recruitment assays (unpublished data). In a bioluminescence resonance energy transfer–based binding assay, activation of GPR1 by chemerin did not induce binding of any G protein, but only *β*-arrestin (De Henau et al., 2016). These findings require further investigation: key experiments would exploit technology such as the CellKey system to test chemerin activation of GPR1-transfected and CMKLR1-transfected cells in parallel to compare the response size. In addition, Marchese et al. (1994a) highlight an interesting observation: rat GPR1 has 77% sequence identity with the human receptor, but it has the conserved arginine residue of the DRY motif like other GPCRs. Mouse GPR1 also has the conserved DRY motif. Using rodent GPR1- and human GPR1-transfected cells in the above-mentioned assay would be a valuable comparison to test the effect of the mutation. Worthy of note is that in CMKLR1 the “DRY” motif is “DRC” across species (Figs. 2 and 4). This is a known modification of the G protein–binding motif that does not affect signaling (Rovati et al., 2007).

## IX. Physiologic Roles

### A. Chemerin Receptor 1

#### 1. Immunity

The specific expression pattern of CMKLR1 on dendritic cells and macrophages highlights the key function of this receptor in innate and adaptive immunity. Chemerin-stimulated recruitment of these antigen-presenting cells suggests that this receptor can initiate an early immune response (Mariani and Roncucci, 2015). It is well characterized that chemerin is a proinflammatory chemotactic agent that through activation of CMKLR1 causes the transmigration of dendritic cells and macrophages in humans (Wittamer et al., 2003; Vermi et al., 2005; Zabel et al., 2005b, 2006; Albanesi et al., 2009; Skrzeczyńska-Moncznik et al., 2009b; De Palma et al., 2011) and mice (Hart and Greaves, 2010; Gonzalvo-Feo et al., 2014). CMKLR1 has been implicated in the development of the immune response by stimulating recruitment of natural killer cells (Parolini et al., 2007; Skrzeczyńska-Moncznik et al., 2009a). A handful of studies in mice has reported that chemerin also exhibits anti-inflammatory effects via CMKLR1. C15 enhanced macrophage phagocytosis, leading to improved microbial particle clearance and apoptotic neutrophil ingestion (Cash et al., 2010) and increased macrophage adhesion capacity (Hart and Greaves, 2010). RvE1 enhanced human macrophage phagocytosis via CMKLR1 (Ohira et al., 2010), and similarly, it has been shown in mice in vivo (Schwab et al., 2007) and in vitro (Hong et al., 2008). In addition, chemerin reduced neutrophil inflammation and inhibited the release of inflammatory cytokines in a mouse model of lung inflammation (Luangsay et al., 2009). The differing proinflammatory and anti-inflammatory effects of CMKLR1 could be due to activation of the receptor by different isoforms of chemerin, or it could suggest that the chemerin/CMKLR1 signaling pathway can activate and limit its actions depending on the environment it is mediating.

#### 2. Antibacterial/Antimicrobial Agent

Based on the structure of chemerin, it is predicted to belong to the cathelicidin/cystatin family of proteins, which includes the antibacterial polypeptides cathelicidins, suggesting that chemerin could play a role in host defense. Chemerin is highly abundant in human skin and has been identified as an antimicrobial and antibacterial reagent, significantly inhibiting bacteria growth in vitro (Kulig et al., 2011; Banas et al., 2013). Keratinocytes respond to microbial stimuli by producing chemerin. The epidermis of the skin responds to some bacterial strains through upregulation of CMKLR1, which is likely to respond to chemerin in an autocrine manner to give chemerin its antimicrobial functionality (Banas et al., 2015).

#### 3. Adipogenesis and Energy Metabolism

In 2007, the important discovery of chemerin and CMKLR1 expression in adipose tissue (Goralski et al., 2007) changed our comprehension of chemerin biology. Chemerin and CMKLR1 have been found to be important regulators of adipogenesis. Expression of both increases throughout maturation of periadipocytes in humans (Roh et al., 2007; Sell et al., 2009), mice (Goralski et al., 2007), and cows (Suzuki et al., 2012). Knockdown of chemerin or CMKLR1 by ribonucleic acid interference inhibits adipogenesis in human and mouse periadipocytes (Goralski et al., 2007) and human bone marrow stromal cells, which can also differentiate into adipocytes (Muruganandan et al., 2010). Maturation of adipocytes leads to an increase in secretion of bioactive chemerin; therefore, adipocytes are both producing and targeted by chemerin signaling (Goralski and Sinal, 2009).

In mouse 3T3-L1 adipocytes, chemerin potentiated insulin-stimulated glucose uptake (Takahashi et al., 2008), and chemerin knockdown decreased insulin-stimulated glucose uptake (Goralski and Sinal, 2009). In vivo, a metabolically stable analog of C9 caused a reduction of free fatty acids in the plasma in fasted mice, suggested to be due to the antilipolytic activity of C9 (Shimamura et al., 2009), although it could also be through potentiation of the insulin signaling. In contrast, chemerin impaired insulin signaling and blocked insulin-stimulated glucose uptake by human skeletal muscle cells (Sell et al., 2009) and induced insulin resistance in rat cardiomyocytes (Zhang et al., 2014). Although it has not been explicitly stated which chemerin receptor is mediating the actions of chemerin in insulin signaling and adipocyte metabolism, these studies imply that it is through CMKLR1. However, Goralski and Sinal, (2009) report that CMKLR1 knockdown had no effect on insulin-stimulated glucose uptake, and the knockout mouse studies are inconclusive (see *Section XI. A. Chemerin Receptor 1*); therefore, further study is required to identify which receptor is mediating chemerin-induced alterations in energy metabolism.

#### 4. Cardiovascular System

Chemerin has been reported to have roles in the human cardiovascular system. Chemerin activation of CMKLR1 on smooth muscle cells caused a potent contraction in vitro in resistance vessels from humans (Kennedy et al., 2016) and rats, but could not be replicated in mice (Watts et al., 2013). This translated into an increase in blood pressure in vivo in healthy rats via CMKLR1 (Kennedy et al., 2016) and in mice (Kunimoto et al., 2015). A single nucleotide polymorphism (SNP) in the *EIDL3* gene was found to be strongly associated with serum chemerin levels, and this gene is known for its role in angiogenesis (Bozaoglu et al., 2010). Subsequent in vitro studies using human endothelial cells showed that chemerin induced the growth of capillary-like structures in a similar manner to vascular endothelial growth factor (Bozaoglu et al., 2010). Also, chemerin activated key MAPK and Akt signaling pathways and stimulated matrix metalloproteinase gelatinolytic activity to induce cell migration and angiogenesis (Kaur et al., 2010). Although in both cases it has not been identified which receptor is responsible for the angiogenic properties of chemerin, Kaur et al. (2010) imply that it is via CMKLR1 due to its expression in human endothelial cells. Chemerin-induced angiogenesis has also been reported in gastric cancers (Wang et al., 2014) and rat decidua endothelial cells (Carlino et al., 2012).

### B. Chemerin Receptor 2

There are currently no reported physiologic roles of GPR1. Functional studies with receptor knockout mice have identified potential roles in pathophysiology.

## X. Pathophysiological Roles

### A. Chemerin Receptor 1

#### 1. Inflammation

Chemerin is abundant in a diverse range of inflammatory fluids, including ascitic fluids from ovarian or liver cancer, or ovary hyperstimulation syndrome, as well as synovial fluids from arthritic patients. This suggests that the chemerin/CMKLR1 system is involved in many pathologies that have a proinflammatory element (Wittamer et al., 2003). Retinoid acid–activated human endothelial cells (representing inflamed endothelial cells seen in inflammatory diseases) promoted myeloid and plasmacytoid dendritic cell translocation across an endothelial monolayer through chemerin production and CMKLR1 activation (Gonzalvo-Feo et al., 2014). This has been reported in psoriasis (Vermi et al., 2005; Albanesi et al., 2009; Skrzeczyńska-Moncznik et al., 2009b), lupus nephritis (De Palma et al., 2011), and multiple sclerosis (Lande et al., 2008). Chemerin not only initiates its own inflammatory response by recruitment of immune cells, but can also upregulate production of other inflammatory mediators via CMKLR1. One example is increased production of interleukin-6, chemokine (C-C motif) ligand 2, and matrix metalloproteinase 3 from fibroblast-like synoviocytes by chemerin in rheumatoid arthritis (Kaneko et al., 2011). Animal models using CMKLR1-deficient mice (see *Section XI. A. Chemerin Receptor 1*) are in agreement that chemerin/CMKLR1 have a role in the modulation of inflammatory diseases, and, similar to in vitro studies, chemerin exhibits both pro- and anti-inflammatory responses.

#### 2. Obesity

It has been well characterized that circulating chemerin levels are higher in patients with obesity (Bozaoglu et al., 2007; Sell et al., 2009; Stefanov et al., 2014), and chemerin and CMKLR1 expression is upregulated in adipose tissue from obese subjects (Catalán et al., 2013). Similarly, chemerin and CMKLR1 levels are higher in mice fed on a high-fat diet (Roh et al., 2007) and mouse models of obesity (Ernst et al., 2010; Parlee et al., 2010). Chemerin is well characterized in its role in adipogenesis and energy metabolism (see *Section IX. A. 3. Adipogenesis and Energy Metabolism*), and it is through these mechanisms that the link between the adipokine and obesity is explained. Another important observation is that proinflammatory mediator, TNF-*α*, upregulated chemerin expression in periadipocytes from humans (Catalán et al., 2013), mice (Parlee et al., 2010), and cows (Suzuki et al., 2012), suggesting that the chronic low levels of inflammation seen in obesity could enhance further the autocrine chemerin signaling. Further discussion on this topic can be found in a review by Roman et al. (2012).

#### 3. Cardiovascular Disease

A novel role of chemerin and CMKLR1 in cardiovascular disease is emerging, both as a consequence of this signaling pathway’s known roles in obesity/metabolic syndrome and inflammation (as outlined above) as well as direct actions on the cardiovascular system.

The implications of CMKLR1 in obesity increase the risk of these patients developing cardiovascular disease. In addition, hypertensive patients have significantly higher plasma concentrations of chemerin compared with healthy controls, and levels of chemerin positively correlate with body mass index and blood pressure (Yang et al., 2010). The finding that chemerin caused a significant increase in blood pressure in vivo in normotensive rats (Kennedy et al., 2016) suggests that in conditions such as obesity, in which chemerin levels are chronically upregulated (Stejskal et al., 2008; Dong et al., 2011; Lin et al., 2012; Wang et al., 2013; Stefanov et al., 2014), this could lead to the development of hypertension by direct activation of CMKLR1 on the vascular smooth muscle. There has been a proposal put forward by Ferland and Watts (2015) for a further role of chemerin in the pathology of hypertension through its ability to activate dendritic cells (most likely via CMKLR1) after injury and stimulate an immune axis of hypertension, but as yet no experimental evidence of this.

Chemerin and its receptor could be involved in atherosclerosis. Chemerin and CMKLR1 expression were present in atherosclerotic plaques (Kostopoulos et al., 2014), and aortic and coronary atherosclerosis positively correlates with chemerin expression in the periaortic or pericoronary adipose tissue (Spiroglou et al., 2010). Similarly, serum chemerin levels reflect the extent of coronary atherosclerosis in patients (Xiaotao et al., 2012). The exact mechanisms of chemerin/CMKLR1 signaling in atherosclerosis have yet to be investigated, so further research needs to be carried out. Some ideas that are currently being considered are that CMKLR1 could initiate the disease through vascular injury, either by abnormal contractile function (Ferland and Watts, 2015) or decreased nitric oxide/cGMP signaling (Neves et al., 2014), which could be a result of downregulation of chemerin/CMKLR1 seen in rat endothelial cells treated with atherogenic factors (Zhao et al., 2013). The proinflammatory nature of chemerin could induce chemotaxis of CMKLR1-expressing dendritic cells, macrophages, and natural killer cells contributing to the progression of the disease (Rhee, 2011), as could its known roles in adipogenesis and lipid metabolism (Goralski et al., 2007) and angiogenesis (Kaur et al., 2010).

Some studies have reported anti-inflammatory roles of CMKLR1 that are cardioprotective. In mice, C15 treatment prior to acute myocardial infraction inhibited neutrophil infiltration and heart damage (Cash et al., 2013). RvE1 blocks human vascular smooth muscle cell migration, a pivotal stage in the onset of atherosclerosis (Ho et al., 2010). Activation of non-neuronal muscarinic receptor enhances chemerin/CMKLR1 signaling in a dysfunctional endothelium to restore function (Zhao et al., 2013).

In rat aorta, chemerin increases vascular constrictor responses to phenylephrine and endothelin-1 (Lobato et al., 2012), and in mouse cardiomyocytes chemerin induced apoptosis with chemerin levels dependent on the environment, upregulated by proinflammatory TNF-*α* and downregulated by insulin (Rodríguez-Penas et al., 2015). Again, it has yet to be determined which receptor is mediating these effects, but they could also be means by which chemerin/CMKLR1 contribute to cardiovascular disease.

#### 4. Other Roles

There are many reported single-nucleotide variations in the human CMKLR1 gene, but as yet only one has been associated with pathophysiology. The SNP rs1878022 located within an intron in the CMKLR1 gene on chromosome12q23.3 was statistically significantly associated with decreased overall survival in advanced stage patients with nonsmall cell lung cancer undergoing platinum-based chemotherapy (Wu et al., 2011). This suggests that CMKLR1 could have a role in cancer, and in vitro studies are in agreement. Chemerin stimulates cell invasion of human esophageal squamous cancer cell (Kumar et al., 2016), gastric cancer cells (Wang et al., 2014), and mesenchymal stromal cells (Kumar et al., 2014). The unclear picture surrounding the role of chemerin in inflammation is apparent in cancer as well. In mice, coculturing macrophages with tumor cells caused an upregulation of CMKLR1, and, when treated with chemerin, there was increased expression of proinflammatory mediators (Rama et al., 2011). However, another report found that in melanoma, chemerin inhibited tumor growth in vivo by recruiting CMKLR1-expressing natural killer cells (Pachynski et al., 2012).

CMKLR1 has been further implicated in other diseases through limited studies. One study has shown that CMKLR1 mRNA was upregulated in brain tissue of patients with Alzheimer’s disease, proposing that CMKLR1 has a role in chemotaxis of microglia cells and uptake of inflammatory mediator A*β*42 (Peng et al., 2015). CMKLR1 was found to be essential for myogenic differentiation in mouse C2C12 cells, and this was consistent with *CMKLR1*-deficient mice having a subtle skeletal muscle deficit (Issa et al., 2012).

### B. Chemerin Receptor 2

#### 1. Human Immunodeficiency Virus

The only known role of human GPR1 is in human immunodeficiency virus (HIV) replication. GPR1 acts as a coreceptor facilitating the replication of HIV-1 and HIV-2 in brain-derived cells (Edinger et al., 1997; Shimizu et al., 2009) and HIV/simian immunodeficiency virus in mesangial cells of the glomerulus (Tokizawa et al., 2000). It has also been identified as a coreceptor to HIV strains derived from brain and blood samples from patients with AIDS (Ohagen et al., 2003). Using the N-terminal extracellular region of GPR1, Jinno-Oue et al. (2005) synthesized a peptide, GPR1ntP-(1–27), based on the N terminus of human GPR1, that inhibited the infection of different strains of HIV. This effect was not specific to HIV strains that required GPR1 as a coreceptor, and so it is believed that GPR1ntP-(1–27) interacts directly with intact HIV virions to prevent binding and therefore entry into host cells.

#### 2. Other Roles

A knockout mouse study has identified that GPR1 is involved in the regulation of glucose homeostasis (Rourke et al., 2014), suggesting that this novel chemerin receptor could have a comparable role to CMKLR1 in metabolic disorders (see *Section X. A. 2. Obesity*).

Also in concordance with the emerging roles of CMKLR1 in the cardiovascular system, GPR1 has been linked to cardiovascular disease. The Medical Research Council’s British Genetics of Hypertension study identified GPR1 as one of the candidate genes that corresponded to a region of interest in the genome-wide mapping of human loci for essential hypertension (Caulfield et al., 2003). GPR1 mRNA was found to be regulated during the transformation of contractile and quiescent smooth muscle cells to the proliferative, migratory, and synthetic state, a pivotal stage in the onset and progression of atherosclerosis (Karagiannis et al., 2013).

Similarly, in normal human oral keratinocytes, GPR1 mRNA was found to be significantly upregulated during senescence compared with the proliferating phenotype (Kang et al., 2003), suggesting that GPR1 could play a more general role in cell transformations.

Most recently, a novel role of GPR1 was reported in progesterone synthesis in mice. Chemerin suppressed progesterone production in cultured follicle and corpus luteum and prostaglandin F2*α*-activated caspase-3, resulting in a subsequent reduction in progesterone secretion, both of which were mediated by this receptor (Yang et al., 2016). Likewise, although the receptor(s) responsible has yet to be identified, chemerin inhibited production of progesterone and estrodial in human (Reverchon et al., 2012) and rat (Wang et al., 2012) granulosa cells and testosterone in rat testis (Li et al., 2014b), suggesting a role for chemerin in the regulation of gonodal steriogenesis.

The identification of these second active signaling chemerin receptors means that it is now imperative to pharmacologically characterize the target of novel functions of chemerin. The current studies, which hypothesize that the effect is through one receptor based only on receptor distribution (at times when both receptor distributions have not been studied), need to be repeated in the presence of a selective antagonist to confirm the target. Presently, there is overlap in function of GPR1 and CMKLR1 in adipogenesis and lipid metabolism, the cardiovascular system, and reproductive biology. The contradictory literature around the function of CMKLR1 particularly in the adipose tissue could be due to influences of chemerin signaling at GPR1, advocating further the need for a GPR1 antagonist to be developed.

## XI. Genetically Modified Animals

### A. Chemerin Receptor 1

Disruption of the *CMKLR1* gene in mice resulted in viable homozygous and heterozygous offspring that displayed no differences in breeding, growth, and survival compared with WT under physiologic conditions (Graham et al., 2009; Luangsay et al., 2009).

Knockout models have exemplified the role of CMKLR1 in inflammation and are in agreement with the in vitro studies that chemerin through CMKLR1 has the ability to induce both pro- and anti-inflammatory effects. Compared with WT controls, *CMKLR1^−/−^* mice had significantly reduced clinical signs to the dihydrotestosterone-induced polycystic ovary syndrome (Tang et al., 2016), experimental autoimmune encephalomyelitis (Graham et al., 2009), and chronic obstructive pulmonary disease (Demoor et al., 2011). The *CMKLR1^−/−^* mice were shown to have a slower development of clinical symptoms of irritable bowel syndrome, although ultimately developed similar levels of inflammation and illness as WT (Dranse et al., 2015). *CMKLR1^−/−^* mice gave a similar inflammatory response, with equivalent neutrophil and monocyte recruitment to WT mice in zymosan-induced peritonitis (Cash et al., 2008). However, *CMKLR1*-deficient mice had an increased inflammatory response in lipopolysaccharide-induced acute lung inflammation (Luangsay et al., 2009) and had delayed clearance of the virus and a higher mortality, in the pneumonia virus of mice model (Bondue et al., 2011a), as a result of the loss of the anti-inflammatory pathways involving CMKLR1.

Chemerin has been shown to have antimicrobial properties; chemerin-deficient mice topically infected with *Staphylococcus aureus* had 10 times higher bacterial levels after 24 hours compared with WT (Banas et al., 2013). The study implies, due to an increased expression seen in the skin, that chemerin is acting through CMKLR1, although the exact mechanism has not been identified.

The *CMKLR1* knockout mice have highlighted the potential role of CMKLR1 in metabolic syndrome and obesity, although findings are not consistent. Ernst et al. (2012) report that *CMKLR1^−/−^* mice had a lower food consumption, leading to decreased total body mass and percent body fat; they had reduced inflammatory responses, including decreased TNF-*α* and interleukin-6 levels in adipose tissue, decreased hepatic dendritic cell inflammation, and increased natural killer cells in adipose tissue, and were glucose intolerant compared with WT mice. These observations were found whether the mice were fed on a normal or a high-fat diet. However, others identified that *CMKLR1^−/−^* mice only had a mild tendency to obesity (Wargent et al., 2015) but did exacerbate impaired glucose homeostasis and insulin resistance (Huang et al., 2016) when fed on a high-fat diet. In contrast, some studies describe no effect on body weight and no difference in insulin resistance in *CMKLR1^−/−^* mice even when fed on a high-fat diet (Rouger et al., 2013; Gruben et al., 2014), and thus when *CMKLR1*-deficient bone marrow was transplanted into a mouse model of nonalcohol fatty liver disease (low-density lipid receptor knockout), there was no difference in liver pathology or insulin resistance (Gruben et al., 2014). Although adipocyte differentiation was unaffected in *CMKLR1^−/−^* mice, GPD1 expression, a lipogenic marker, was elevated, suggesting CMKLR1 could have a role in adipocyte metabolism (Rouger et al., 2013).

*CMKLR1^−/−^* mice on a high-fat diet also had no response when exposed to cold conditions, whereas WT mice had a significant decrease in body mass and improved glucose intolerance and insulin resistance, suggesting a role of CMKLR1 in regulating thermogenesis (Huang et al., 2016).

### B. Chemerin Receptor 2

There has currently only been one study carried out using *GPR1*-deficient mice. Disruption of the *GPR1* gene in mice resulted in viable homozygous and heterozygous offspring that displayed no differences in breeding, growth, and survival compared with WT under physiologic conditions. However, Rourke et al. (2014) report that when fed on a high-fat diet, *GPR1* knockout mice develop heightened glucose intolerance, compared with WT, with no effect on body weight, percent body fat, or energy expenditure due to consumption of significantly less food. When tested for pyruvic acid tolerance, *GPR1^−/−^* exhibited reduced glucose-stimulated insulin levels, leading to an increase in blood glucose levels. Similar to CMKLR1, this suggests that GPR1 may also be involved in adipogenesis and glucose homeostasis.

Although no SNPs reported in the *GPR1* or *CMKLR1* gene implicate them with obesity and metabolic syndrome, there is a positive correlation found between common genetic variants in the *RARRES2* gene (SNP rs17173608 and rs10278590) with increased visceral adiposity (Müssig et al., 2009). Similarly, chemerin-deficient mice showed impaired glucose-dependent insulin secretion, and chemerin transgenic mice had improved glucose-dependent insulin secretion and glucose tolerance (Takahashi et al., 2011). However, the authors did not determine through which receptor chemerin was functioning. A must-do study is to knock out both *GPR1* and *CMKLR1* in mice to investigate whether it is through both receptors that chemerin exerts its effects. Comparisons of responses in double-knockout mice with chemerin-deficient and single-receptor knockout mice would be insightful. This strategy could be employed in areas where both receptors appear to have similar physiology.

There are reported SNPs in the human GPR1 gene, with one, rs16838070, shown to be associated with increased risk of developing late-onset Alzheimer’s disease (Chaudhry et al., 2015). It could therefore be important to investigate a role for GPR1 in Alzheimer’s disease, and it might be of interest to explore whether A*β*42, a proposed ligand for CMKLR1, which is crucially involved in the pathogenesis of Alzheimer’s disease, binds and functions through GPR1.

## Abbreviations

A*β*42: amyloid-*β* peptide 42
CHO: Chinese hamster ovary
CMKLR1: chemokine-like receptor 1
GPCR: G protein–coupled receptor
GPR1: G protein–coupled receptor 1
HIV: human immunodeficiency virus
MAPK: mitogen-activated protein kinase
*α*-NETA: 2-(anaphthoyl) ethyltrimethylammonium iodide
PTX: pertussis toxin
RvE1: resolvin E1
SNP: single nucleotide polymorphism
TNF: tumor necrosis factor
WT: wild-type

## References

[B1] Albanesi C Scarponi C Pallotta S Daniele R Bosisio D Madonna S Fortugno P Gonzalvo-Feo S Franssen JD Parmentier M (2009) Chemerin expression marks early psoriatic skin lesions and correlates with plasmacytoid dendritic cell recruitment. J Exp Med 206:249–258. 19114666 10.1084/jem.20080129 PMC2626680

[B2] Arita M Bianchini F Aliberti J Sher A Chiang N Hong S Yang R Petasis NA Serhan CN (2005) Stereochemical assignment, antiinflammatory properties, and receptor for the omega-3 lipid mediator resolvin E1. J Exp Med 201:713–722. 15753205 10.1084/jem.20042031 PMC2212834

[B3] Arita M Ohira T Sun YP Elangovan S Chiang N Serhan CN (2007) Resolvin E1 selectively interacts with leukotriene B4 receptor BLT1 and ChemR23 to regulate inflammation. J Immunol 178:3912–3917. 17339491 10.4049/jimmunol.178.6.3912

[B4] Bachelerie F Graham GJ Locati M Mantovani A Murphy PM Nibbs R Rot A Sozzani S Thelen M (2015) An atypical addition to the chemokine receptor nomenclature: IUPHAR Review 15. Br J Pharmacol 172:3945–3949. 25958743 10.1111/bph.13182 PMC4543604

[B5] Bäck M Powell WS Dahlén SE Drazen JM Evans JF Serhan CN Shimizu T Yokomizo T Rovati GE (2014) Update on leukotriene, lipoxin and oxoeicosanoid receptors: IUPHAR Review 7. Br J Pharmacol 171:3551–3574. 24588652 10.1111/bph.12665 PMC4128057

[B6] Banas M Zabieglo K Kasetty G Kapinska-Mrowiecka M Borowczyk J Drukala J Murzyn K Zabel BA Butcher EC Schroeder JM (2013) Chemerin is an antimicrobial agent in human epidermis. PLoS One 8:e58709. 23527010 10.1371/journal.pone.0058709 PMC3604073

[B7] Banas M Zegar A Kwitniewski M Zabieglo K Marczynska J Kapinska-Mrowiecka M LaJevic M Zabel BA Cichy J (2015) The expression and regulation of chemerin in the epidermis. PLoS One 10:e0117830. 25659101 10.1371/journal.pone.0117830 PMC4320080

[B8] Barnea G Strapps W Herrada G Berman Y Ong J Kloss B Axel R Lee KJ (2008) The genetic design of signaling cascades to record receptor activation. Proc Natl Acad Sci USA 105:64–69. 18165312 10.1073/pnas.0710487105 PMC2224232

[B9] Bondue B De Henau O Luangsay S Devosse T de Nadaï P Springael JY Parmentier M Vosters O (2012) The chemerin/ChemR23 system does not affect the pro-inflammatory response of mouse and human macrophages ex vivo. PLoS One 7:e40043. 22768214 10.1371/journal.pone.0040043 PMC3386906

[B10] Bondue B Vosters O de Nadai P Glineur S De Henau O Luangsay S Van Gool F Communi D De Vuyst P Desmecht D (2011 a) ChemR23 dampens lung inflammation and enhances anti-viral immunity in a mouse model of acute viral pneumonia. PLoS Pathog 7:e1002358. 22072972 10.1371/journal.ppat.1002358 PMC3207933

[B11] Bondue B Wittamer V Parmentier M (2011 b) Chemerin and its receptors in leukocyte trafficking, inflammation and metabolism. Cytokine Growth Factor Rev 22:331–338. 22119008 10.1016/j.cytogfr.2011.11.004

[B12] Bozaoglu K Bolton K McMillan J Zimmet P Jowett J Collier G Walder K Segal D (2007) Chemerin is a novel adipokine associated with obesity and metabolic syndrome. Endocrinology 148:4687–4694. 17640997 10.1210/en.2007-0175

[B13] Bozaoglu K Curran JE Stocker CJ Zaibi MS Segal D Konstantopoulos N Morrison S Carless M Dyer TD Cole SA (2010) Chemerin, a novel adipokine in the regulation of angiogenesis. J Clin Endocrinol Metab 95:2476–2485. 20237162 10.1210/jc.2010-0042 PMC2869547

[B14] Carlino C Trotta E Stabile H Morrone S Bulla R Soriani A Iannitto ML Agostinis C Mocci C Minozzi M (2012) Chemerin regulates NK cell accumulation and endothelial cell morphogenesis in the decidua during early pregnancy. J Clin Endocrinol Metab 97:3603–3612. 22791765 10.1210/jc.2012-1102 PMC3462933

[B146] Cash JL Bass MD Campbell J Barnes M Kubes P Martin P (2014) Resolution mediator chemerin15 reprograms the wound microenvironment to promote repair and reduce scarring. Curr Biol 24:1406–1414. 24881877 10.1016/j.cub.2014.05.006 PMC4064685

[B15] Cash JL Bena S Headland SE McArthur S Brancaleone V Perretti M (2013) Chemerin15 inhibits neutrophil-mediated vascular inflammation and myocardial ischemia-reperfusion injury through ChemR23. EMBO Rep 14:999–1007. 23999103 10.1038/embor.2013.138 PMC3818079

[B16] Cash JL Christian AR Greaves DR (2010) Chemerin peptides promote phagocytosis in a ChemR23- and Syk-dependent manner. J Immunol 184:5315–5324. 20363975 10.4049/jimmunol.0903378 PMC4237835

[B17] Cash JL Hart R Russ A Dixon JP Colledge WH Doran J Hendrick AG Carlton MB Greaves DR (2008) Synthetic chemerin-derived peptides suppress inflammation through ChemR23. J Exp Med 205:767–775. 18391062 10.1084/jem.20071601 PMC2292217

[B18] Catalán V Gómez-Ambrosi J Rodríguez A Ramírez B Rotellar F Valentí V Silva C Gil MJ Salvador J Frühbeck G (2013) Increased levels of chemerin and its receptor, chemokine-like receptor-1, in obesity are related to inflammation: tumor necrosis factor-α stimulates mRNA levels of chemerin in visceral adipocytes from obese patients. Surg Obes Relat Dis 9:306–314. 22154272 10.1016/j.soard.2011.11.001

[B19] Caulfield M Munroe P Pembroke J Samani N Dominiczak A Brown M Benjamin N Webster J Ratcliffe P O’Shea S MRC British Genetics of Hypertension Study (2003) Genome-wide mapping of human loci for essential hypertension. Lancet 361:2118–2123. 12826435 10.1016/S0140-6736(03)13722-1

[B20] Chaudhry M Wang X Bamne MN Hasnain S Demirci FY Lopez OL Kamboh MI (2015) Genetic variation in imprinted genes is associated with risk of late-onset Alzheimer’s disease. J Alzheimers Dis 44:989–994. 25391383 10.3233/JAD-142106 PMC4324355

[B147] Clark-Lewis I Kim KS Rajarathnam K Gong JH Dewald B Moser B Baggiolini M Sykes BD (1995) Structure-activity relationships of chemokines. J Leukoc Biol 57:703–711. 7759949 10.1002/jlb.57.5.703

[B21] Davenport AP Alexander SP Sharman JL Pawson AJ Benson HE Monaghan AE Liew WC Mpamhanga CP Bonner TI Neubig RR (2013) International Union of Basic and Clinical Pharmacology. LXXXVIII. G protein-coupled receptor list: recommendations for new pairings with cognate ligands. Pharmacol Rev 65:967–986. 23686350 10.1124/pr.112.007179 PMC3698937

[B22] De Henau O Degroot GN Imbault V Robert V De Poorter C Mcheik S Galés C Parmentier M Springael JY (2016) Signaling properties of chemerin receptors CMKLR1, GPR1 and CCRL2. PLoS One 11:e0164179. 27716822 10.1371/journal.pone.0164179 PMC5055294

[B23] Demoor T Bracke KR Dupont LL Plantinga M Bondue B Roy MO Lannoy V Lambrecht BN Brusselle GG Joos GF (2011) The role of ChemR23 in the induction and resolution of cigarette smoke-induced inflammation. J Immunol 186:5457–5467. 21430224 10.4049/jimmunol.1003862

[B24] De Palma G Castellano G Del Prete A Sozzani S Fiore N Loverre A Parmentier M Gesualdo L Grandaliano G Schena FP (2011) The possible role of ChemR23/Chemerin axis in the recruitment of dendritic cells in lupus nephritis. Kidney Int 79:1228–1235. 21346723 10.1038/ki.2011.32

[B25] de Poorter C Baertsoen K Lannoy V Parmentier M Springael JY (2013) Consequences of ChemR23 heteromerization with the chemokine receptors CXCR4 and CCR7. PLoS One 8:e58075. 23469143 10.1371/journal.pone.0058075 PMC3585228

[B26] Diot M Reverchon M Rame C Froment P Brillard JP Brière S Levêque G Guillaume D Dupont J (2015) Expression of adiponectin, chemerin and visfatin in plasma and different tissues during a laying season in turkeys. Reprod Biol Endocrinol 13:81. 26228641 10.1186/s12958-015-0081-5 PMC4521348

[B27] Dong B Ji W Zhang Y (2011) Elevated serum chemerin levels are associated with the presence of coronary artery disease in patients with metabolic syndrome. Intern Med 50:1093–1097. 21576834 10.2169/internalmedicine.50.5025

[B28] Dranse HJ Rourke JL Stadnyk AW Sinal CJ (2015) Local chemerin levels are positively associated with DSS-induced colitis but constitutive loss of CMKLR1 does not protect against development of colitis. Physiol Rep 3:e12497. 26265756 10.14814/phy2.12497 PMC4562582

[B29] Du XY Zabel BA Myles T Allen SJ Handel TM Lee PP Butcher EC Leung LL (2009) Regulation of chemerin bioactivity by plasma carboxypeptidase N, carboxypeptidase B (activated thrombin-activable fibrinolysis inhibitor), and platelets. J Biol Chem 284:751–758. 19010784 10.1074/jbc.M805000200 PMC2613638

[B30] Edinger AL Mankowski JL Doranz BJ Margulies BJ Lee B Rucker J Sharron M Hoffman TL Berson JF Zink MC (1997) CD4-independent, CCR5-dependent infection of brain capillary endothelial cells by a neurovirulent simian immunodeficiency virus strain. Proc Natl Acad Sci USA 94:14742–14747. 9405683 10.1073/pnas.94.26.14742 PMC25107

[B31] Ernst MC Haidl ID Zúñiga LA Dranse HJ Rourke JL Zabel BA Butcher EC Sinal CJ (2012) Disruption of the chemokine-like receptor-1 (CMKLR1) gene is associated with reduced adiposity and glucose intolerance. Endocrinology 153:672–682. 22186410 10.1210/en.2011-1490 PMC3275396

[B32] Ernst MC Issa M Goralski KB Sinal CJ (2010) Chemerin exacerbates glucose intolerance in mouse models of obesity and diabetes. Endocrinology 151:1998–2007. 20228173 10.1210/en.2009-1098

[B33] Ernst MC Sinal CJ (2010) Chemerin: at the crossroads of inflammation and obesity. Trends Endocrinol Metab 21:660–667. 20817486 10.1016/j.tem.2010.08.001

[B34] Farzan M Choe H Martin K Marcon L Hofmann W Karlsson G Sun Y Barrett P Marchand N Sullivan N (1997) Two orphan seven-transmembrane segment receptors which are expressed in CD4-positive cells support simian immunodeficiency virus infection. J Exp Med 186:405–411. 9236192 10.1084/jem.186.3.405 PMC2198994

[B35] Fatima SS Rehman R Baig M Khan TA (2014) New roles of the multidimensional adipokine: chemerin. Peptides 62:15–20. 25278490 10.1016/j.peptides.2014.09.019

[B36] Ferland DJ Watts SW (2015) Chemerin: a comprehensive review elucidating the need for cardiovascular research. Pharmacol Res 99:351–361. 26211950 10.1016/j.phrs.2015.07.018 PMC4859430

[B37] Fredman G Van Dyke TE Serhan CN (2010) Resolvin E1 regulates adenosine diphosphate activation of human platelets. Arterioscler Thromb Vasc Biol 30:2005–2013. 20702811 10.1161/ATVBAHA.110.209908 PMC2982748

[B38] Gantz I Konda Y Yang YK Miller DE Dierick HA Yamada T (1996) Molecular cloning of a novel receptor (CMKLR1) with homology to the chemotactic factor receptors. Cytogenet Cell Genet 74:286–290. 8976386 10.1159/000134436

[B39] Gonzalvo-Feo S Del Prete A Pruenster M Salvi V Wang L Sironi M Bierschenk S Sperandio M Vecchi A Sozzani S (2014) Endothelial cell-derived chemerin promotes dendritic cell transmigration. J Immunol 192:2366–2373. 24470498 10.4049/jimmunol.1302028

[B40] Goralski KB McCarthy TC Hanniman EA Zabel BA Butcher EC Parlee SD Muruganandan S Sinal CJ (2007) Chemerin, a novel adipokine that regulates adipogenesis and adipocyte metabolism. J Biol Chem 282:28175–28188. 17635925 10.1074/jbc.M700793200

[B41] Goralski KB Sinal CJ (2009) Elucidation of chemerin and chemokine-like receptor-1 function in adipocytes by adenoviral-mediated shRNA knockdown of gene expression. Methods Enzymol 460:289–312. 19446731 10.1016/S0076-6879(09)05214-8

[B42] Gouwy M Struyf S Leutenez L Pörtner N Sozzani S Van Damme J (2014) Chemokines and other GPCR ligands synergize in receptor-mediated migration of monocyte-derived immature and mature dendritic cells. Immunobiology 219:218–229. 24268109 10.1016/j.imbio.2013.10.004

[B43] Gouwy M Struyf S Proost P Van Damme J (2005) Synergy in cytokine and chemokine networks amplifies the inflammatory response. Cytokine Growth Factor Rev 16:561–580. 16023396 10.1016/j.cytogfr.2005.03.005

[B44] Graham KL Zabel BA Loghavi S Zuniga LA Ho PP Sobel RA Butcher EC (2009) Chemokine-like receptor-1 expression by central nervous system-infiltrating leukocytes and involvement in a model of autoimmune demyelinating disease. J Immunol 183:6717–6723. 19864606 10.4049/jimmunol.0803435 PMC2904075

[B45] Graham KL Zhang JV Lewén S Burke TM Dang T Zoudilova M Sobel RA Butcher EC Zabel BA (2014) A novel CMKLR1 small molecule antagonist suppresses CNS autoimmune inflammatory disease. PLoS One 9:e112925. 25437209 10.1371/journal.pone.0112925 PMC4249827

[B46] Gruben N Aparicio Vergara M Kloosterhuis NJ van der Molen H Stoelwinder S Youssef S de Bruin A Delsing DJ Kuivenhoven JA van de Sluis B (2014) Chemokine-like receptor 1 deficiency does not affect the development of insulin resistance and nonalcoholic fatty liver disease in mice. PLoS One 9:e96345. 24781986 10.1371/journal.pone.0096345 PMC4004559

[B47] Hart R Greaves DR (2010) Chemerin contributes to inflammation by promoting macrophage adhesion to VCAM-1 and fibronectin through clustering of VLA-4 and VLA-5. J Immunol 185:3728–3739. 20720202 10.4049/jimmunol.0902154

[B48] Herová M Schmid M Gemperle C Hersberger M (2015) ChemR23, the receptor for chemerin and resolvin E1, is expressed and functional on M1 but not on M2 macrophages. J Immunol 194:2330–2337. 25637017 10.4049/jimmunol.1402166

[B49] Ho KJ Spite M Owens CD Lancero H Kroemer AH Pande R Creager MA Serhan CN Conte MS (2010) Aspirin-triggered lipoxin and resolvin E1 modulate vascular smooth muscle phenotype and correlate with peripheral atherosclerosis. Am J Pathol 177:2116–2123. 20709806 10.2353/ajpath.2010.091082 PMC2947304

[B50] Hong S Porter TF Lu Y Oh SF Pillai PS Serhan CN (2008) Resolvin E1 metabolome in local inactivation during inflammation-resolution. J Immunol 180:3512–3519. 18292578 10.4049/jimmunol.180.5.3512

[B51] Huang C Wang M Ren L Xiang L Chen J Li M Xiao T Ren P Xiong L Zhang JV (2016) CMKLR1 deficiency influences glucose tolerance and thermogenesis in mice on high fat diet. Biochem Biophys Res Commun 473:435–441. 26972253 10.1016/j.bbrc.2016.03.026

[B52] Huang J Zhang J Lei T Chen X Zhang Y Zhou L Yu A Chen Z Zhou R Yang Z (2010) Cloning of porcine chemerin, ChemR23 and GPR1 and their involvement in regulation of lipogenesis. BMB Rep 43:491–498. 20663411 10.5483/bmbrep.2010.43.7.491

[B53] Issa ME Muruganandan S Ernst MC Parlee SD Zabel BA Butcher EC Sinal CJ Goralski KB (2012) Chemokine-like receptor 1 regulates skeletal muscle cell myogenesis. Am J Physiol Cell Physiol 302:C1621–C1631. 22460713 10.1152/ajpcell.00187.2011 PMC3378017

[B54] Jinno-Oue A Shimizu N Soda Y Tanaka A Ohtsuki T Kurosaki D Suzuki Y Hoshino H (2005) The synthetic peptide derived from the NH2-terminal extracellular region of an orphan G protein-coupled receptor, GPR1, preferentially inhibits infection of X4 HIV-1. J Biol Chem 280:30924–30934. 15919664 10.1074/jbc.M500195200

[B55] Kaneko K Miyabe Y Takayasu A Fukuda S Miyabe C Ebisawa M Yokoyama W Watanabe K Imai T Muramoto K (2011) Chemerin activates fibroblast-like synoviocytes in patients with rheumatoid arthritis. Arthritis Res Ther 13:R158. 21959042 10.1186/ar3475 PMC3308089

[B56] Kang MK Kameta A Shin KH Baluda MA Kim HR Park NH (2003) Senescence-associated genes in normal human oral keratinocytes. Exp Cell Res 287:272–281. 12837283 10.1016/s0014-4827(03)00061-2

[B57] Karagiannis GS Weile J Bader GD Minta J (2013) Integrative pathway dissection of molecular mechanisms of moxLDL-induced vascular smooth muscle phenotype transformation. BMC Cardiovasc Disord 13:4. 23324130 10.1186/1471-2261-13-4 PMC3556327

[B58] Kaur J Adya R Tan BK Chen J Randeva HS (2010) Identification of chemerin receptor (ChemR23) in human endothelial cells: chemerin-induced endothelial angiogenesis. Biochem Biophys Res Commun 391:1762–1768. 20044979 10.1016/j.bbrc.2009.12.150

[B59] Kennedy AJ Yang P Read C Kuc RE Yang L Taylor EJA Taylor CW Maguire JJ Davenport AP (2016) Chemerin elicits potent constrictor actions via chemokine-like receptor 1 (CMKLR1), not G-protein-coupled receptor 1 (GPR1), in human and rat vasculature. J Am Heart Assoc 5:e004421. 27742615 10.1161/JAHA.116.004421 PMC5121526

[B60] Kostopoulos CG Spiroglou SG Varakis JN Apostolakis E Papadaki HH (2014) Chemerin and CMKLR1 expression in human arteries and periadventitial fat: a possible role for local chemerin in atherosclerosis? BMC Cardiovasc Disord 14:56. 24779513 10.1186/1471-2261-14-56 PMC4022413

[B61] Krishnamoorthy S Recchiuti A Chiang N Yacoubian S Lee CH Yang R Petasis NA Serhan CN (2010) Resolvin D1 binds human phagocytes with evidence for proresolving receptors. Proc Natl Acad Sci USA 107:1660–1665. 20080636 10.1073/pnas.0907342107 PMC2824371

[B62] Kulig P Kantyka T Zabel BA Banas M Chyra A Stefanska A Tu H Allen SJ Handel TM Kozik A (2011) Regulation of chemerin chemoattractant and antibacterial activity by human cysteine cathepsins. J Immunol 187:1403–1410. 21715684 10.4049/jimmunol.1002352 PMC3140563

[B63] Kumar JD Holmberg C Kandola S Steele I Hegyi P Tiszlavicz L Jenkins R Beynon RJ Peeney D Giger OT (2014) Increased expression of chemerin in squamous esophageal cancer myofibroblasts and role in recruitment of mesenchymal stromal cells. PLoS One 9:e104877. 25127029 10.1371/journal.pone.0104877 PMC4134237

[B64] Kumar JD Kandola S Tiszlavicz L Reisz Z Dockray GJ Varro A (2016) The role of chemerin and ChemR23 in stimulating the invasion of squamous oesophageal cancer cells. Br J Cancer 114:1152–1159. 27092781 10.1038/bjc.2016.93 PMC4865978

[B65] Kunimoto H Kazama K Takai M Oda M Okada M Yamawaki H (2015) Chemerin promotes the proliferation and migration of vascular smooth muscle and increases mouse blood pressure. Am J Physiol Heart Circ Physiol 309:H1017–H1028. 26254337 10.1152/ajpheart.00820.2014

[B66] Lande R Gafa V Serafini B Giacomini E Visconti A Remoli ME Severa M Parmentier M Ristori G Salvetti M (2008) Plasmacytoid dendritic cells in multiple sclerosis: intracerebral recruitment and impaired maturation in response to interferon-beta. J Neuropathol Exp Neurol 67:388–401. 18431257 10.1097/NEN.0b013e31816fc975

[B67] Li L Huang C Zhang X Wang J Ma P Liu Y Xiao T Zabel BA Zhang JV (2014 a) Chemerin-derived peptide C-20 suppressed gonadal steroidogenesis. Am J Reprod Immunol 71:265–277. 24506805 10.1111/aji.12164 PMC4092037

[B68] Li L Ma P Huang C Liu Y Zhang Y Gao C Xiao T Ren PG Zabel BA Zhang JV (2014 b) Expression of chemerin and its receptors in rat testes and its action on testosterone secretion. J Endocrinol 220:155–163. 24301613 10.1530/JOE-13-0275 PMC3932185

[B69] Lin X Tang X Jiang Q Liu Q Lin Z Lin J Chen L Hong H (2012) Elevated serum chemerin levels are associated with the presence of coronary artery disease in patients with type 2 diabetes. Clin Lab 58:539–544. 22783586

[B70] Lobato NS Neves KB Filgueira FP Fortes ZB Carvalho MH Webb RC Oliveira AM Tostes RC (2012) The adipokine chemerin augments vascular reactivity to contractile stimuli via activation of the MEK-ERK1/2 pathway. Life Sci 91:600–606. 22521290 10.1016/j.lfs.2012.04.013

[B71] Luangsay S Wittamer V Bondue B De Henau O Rouger L Brait M Franssen JD de Nadai P Huaux F Parmentier M (2009) Mouse ChemR23 is expressed in dendritic cell subsets and macrophages, and mediates an anti-inflammatory activity of chemerin in a lung disease model. J Immunol 183:6489–6499. 19841182 10.4049/jimmunol.0901037

[B72] Marchese A Cheng R Lee MC Porter CA Heiber M Goodman M George SR O’Dowd BF (1994 a) Mapping studies of two G protein-coupled receptor genes: an amino acid difference may confer a functional variation between a human and rodent receptor. Biochem Biophys Res Commun 205:1952–1958. 7811287 10.1006/bbrc.1994.2899

[B73] Marchese A Docherty JM Nguyen T Heiber M Cheng R Heng HH Tsui LC Shi X George SR O’Dowd BF (1994 b) Cloning of human genes encoding novel G protein-coupled receptors. Genomics 23:609–618. 7851889 10.1006/geno.1994.1549

[B74] Mariani F Roncucci L (2015) Chemerin/chemR23 axis in inflammation onset and resolution. Inflamm Res 64:85–95. 25548799 10.1007/s00011-014-0792-7

[B75] Mattern A Zellmann T Beck-Sickinger AG (2014) Processing, signaling, and physiological function of chemerin. IUBMB Life 66:19–26. 24446308 10.1002/iub.1242

[B76] Meder W Wendland M Busmann A Kutzleb C Spodsberg N John H Richter R Schleuder D Meyer M Forssmann WG (2003) Characterization of human circulating TIG2 as a ligand for the orphan receptor ChemR23. FEBS Lett 555:495–499. 14675762 10.1016/s0014-5793(03)01312-7

[B77] Muruganandan S Roman AA Sinal CJ (2010) Role of chemerin/CMKLR1 signaling in adipogenesis and osteoblastogenesis of bone marrow stem cells. J Bone Miner Res 25:222–234. 19929432 10.1359/jbmr.091106

[B78] Müssig K Staiger H Machicao F Thamer C Machann J Schick F Claussen CD Stefan N Fritsche A Häring HU (2009) RARRES2, encoding the novel adipokine chemerin, is a genetic determinant of disproportionate regional body fat distribution: a comparative magnetic resonance imaging study. Metabolism 58:519–524. 19303973 10.1016/j.metabol.2008.11.011

[B79] Nagpal S Patel S Jacobe H DiSepio D Ghosn C Malhotra M Teng M Duvic M Chandraratna RA (1997) Tazarotene-induced gene 2 (TIG2), a novel retinoid-responsive gene in skin. J Invest Dermatol 109:91–95. 9204961 10.1111/1523-1747.ep12276660

[B80] Neves KB Lobato NS Lopes RA Filgueira FP Zanotto CZ Oliveira AM Tostes RC (2014) Chemerin reduces vascular nitric oxide/cGMP signalling in rat aorta: a link to vascular dysfunction in obesity? Clin Sci (Lond) 127:111–122. 24498891 10.1042/CS20130286

[B81] Ohagen A Devitt A Kunstman KJ Gorry PR Rose PP Korber B Taylor J Levy R Murphy RL Wolinsky SM (2003) Genetic and functional analysis of full-length human immunodeficiency virus type 1 env genes derived from brain and blood of patients with AIDS. J Virol 77:12336–12345. 14581570 10.1128/JVI.77.22.12336-12345.2003 PMC254258

[B82] Ohira T Arita M Omori K Recchiuti A Van Dyke TE Serhan CN (2010) Resolvin E1 receptor activation signals phosphorylation and phagocytosis. J Biol Chem 285:3451–3461. 19906641 10.1074/jbc.M109.044131 PMC2823415

[B148] Oldham WM Hamm HE (2008) Heterotrimeric G protein activation by G-protein-coupled receptors. Nat Rev Mol Cell Biol 9:60–71. 18043707 10.1038/nrm2299

[B83] Omasits U Ahrens CH Müller S Wollscheid B (2014) Protter: interactive protein feature visualization and integration with experimental proteomic data. Bioinformatics 30:884–886. 24162465 10.1093/bioinformatics/btt607

[B84] Pachynski RK Zabel BA Kohrt HE Tejeda NM Monnier J Swanson CD Holzer AK Gentles AJ Sperinde GV Edalati A (2012) The chemoattractant chemerin suppresses melanoma by recruiting natural killer cell antitumor defenses. J Exp Med 209:1427–1435. 22753924 10.1084/jem.20112124 PMC3409495

[B85] Parlee SD Ernst MC Muruganandan S Sinal CJ Goralski KB (2010) Serum chemerin levels vary with time of day and are modified by obesity and tumor necrosis factor-α. Endocrinology 151:2590–2602. 20363880 10.1210/en.2009-0794

[B86] Parolini S Santoro A Marcenaro E Luini W Massardi L Facchetti F Communi D Parmentier M Majorana A Sironi M (2007) The role of chemerin in the colocalization of NK and dendritic cell subsets into inflamed tissues. Blood 109:3625–3632. 17202316 10.1182/blood-2006-08-038844

[B87] Peng L Yu Y Liu J Li S He H Cheng N Ye RD (2015) The chemerin receptor CMKLR1 is a functional receptor for amyloid-β peptide. J Alzheimers Dis 43:227–242. 25079809 10.3233/JAD-141227

[B88] Peyrassol X Laeremans T Gouwy M Lahura V Debulpaep M Van Damme J Steyaert J Parmentier M Langer I (2016) Development by genetic immunization of monovalent antibodies (Nanobodies) behaving as antagonists of the human ChemR23 receptor. J Immunol 196:2893–2901. 26864035 10.4049/jimmunol.1500888

[B89] Qu X Zhang X Yao J Song J Nikolic-Paterson DJ Li J (2012) Resolvins E1 and D1 inhibit interstitial fibrosis in the obstructed kidney via inhibition of local fibroblast proliferation. J Pathol 228:506–519. 22610993 10.1002/path.4050

[B90] Rama D Esendagli G Guc D (2011) Expression of chemokine-like receptor 1 (CMKLR1) on J744A.1 macrophages co-cultured with fibroblast and/or tumor cells: modeling the influence of microenvironment. Cell Immunol 271:134–140. 21752353 10.1016/j.cellimm.2011.06.016

[B91] Regard JB Sato IT Coughlin SR (2008) Anatomical profiling of G protein-coupled receptor expression. Cell 135:561–571. 18984166 10.1016/j.cell.2008.08.040 PMC2590943

[B92] Reverchon M Bertoldo MJ Ramé C Froment P Dupont J (2014) CHEMERIN (RARRES2) decreases in vitro granulosa cell steroidogenesis and blocks oocyte meiotic progression in bovine species. Biol Reprod 90:102. 24671882 10.1095/biolreprod.113.117044

[B93] Reverchon M Cornuau M Ramé C Guerif F Royère D Dupont J (2012) Chemerin inhibits IGF-1-induced progesterone and estradiol secretion in human granulosa cells. Hum Reprod 27:1790–1800. 22447628 10.1093/humrep/des089

[B94] Rhee EJ (2011) Chemerin: a novel link between inflammation and atherosclerosis? Diabetes Metab J 35:216–218. 21785740 10.4093/dmj.2011.35.3.216 PMC3138091

[B95] Rodríguez-Penas D Feijóo-Bandín S García-Rúa V Mosquera-Leal A Durán D Varela A Portolés M Roselló-Lletí E Rivera M Diéguez C (2015) The adipokine chemerin induces apoptosis in cardiomyocytes. Cell Physiol Biochem 37:176–192. 26303782 10.1159/000430343

[B96] Roh SG Song SH Choi KC Katoh K Wittamer V Parmentier M Sasaki S (2007) Chemerin--a new adipokine that modulates adipogenesis via its own receptor. Biochem Biophys Res Commun 362:1013–1018. 17767914 10.1016/j.bbrc.2007.08.104

[B97] Roman AA Parlee SD Sinal CJ (2012) Chemerin: a potential endocrine link between obesity and type 2 diabetes. Endocrine 42:243–251. 22610747 10.1007/s12020-012-9698-8

[B98] Rouger L Denis GR Luangsay S Parmentier M (2013) ChemR23 knockout mice display mild obesity but no deficit in adipocyte differentiation. J Endocrinol 219:279–289. 24084834 10.1530/JOE-13-0106

[B99] Rourke JL Dranse HJ Sinal CJ (2015) CMKLR1 and GPR1 mediate chemerin signaling through the RhoA/ROCK pathway. Mol Cell Endocrinol 417:36–51. 26363224 10.1016/j.mce.2015.09.002

[B100] Rourke JL Muruganandan S Dranse HJ McMullen NM Sinal CJ (2014) Gpr1 is an active chemerin receptor influencing glucose homeostasis in obese mice. J Endocrinol 222:201–215. 24895415 10.1530/JOE-14-0069

[B101] Rovati GE Capra V Neubig RR (2007) The highly conserved DRY motif of class A G protein-coupled receptors: beyond the ground state. Mol Pharmacol 71:959–964. 17192495 10.1124/mol.106.029470

[B102] Samson M Edinger AL Stordeur P Rucker J Verhasselt V Sharron M Govaerts C Mollereau C Vassart G Doms RW (1998) ChemR23, a putative chemoattractant receptor, is expressed in monocyte-derived dendritic cells and macrophages and is a coreceptor for SIV and some primary HIV-1 strains. Eur J Immunol 28:1689–1700. 9603476 10.1002/(SICI)1521-4141(199805)28:05<1689::AID-IMMU1689>3.0.CO;2-I

[B103] Schwab JM Chiang N Arita M Serhan CN (2007) Resolvin E1 and protectin D1 activate inflammation-resolution programmes. Nature 447:869–874. 17568749 10.1038/nature05877 PMC2757086

[B104] Sell H Laurencikiene J Taube A Eckardt K Cramer A Horrighs A Arner P Eckel J (2009) Chemerin is a novel adipocyte-derived factor inducing insulin resistance in primary human skeletal muscle cells. Diabetes 58:2731–2740. 19720798 10.2337/db09-0277 PMC2780878

[B105] Shimamura K Matsuda M Miyamoto Y Yoshimoto R Seo T Tokita S (2009) Identification of a stable chemerin analog with potent activity toward ChemR23. Peptides 30:1529–1538. 19540290 10.1016/j.peptides.2009.05.030

[B106] Shimizu N Soda Y Kanbe K Liu HY Jinno A Kitamura T Hoshino H (1999) An orphan G protein-coupled receptor, GPR1, acts as a coreceptor to allow replication of human immunodeficiency virus types 1 and 2 in brain-derived cells. J Virol 73:5231–5239. 10233994 10.1128/jvi.73.6.5231-5239.1999 PMC112576

[B107] Shimizu N Tanaka A Oue A Mori T Ohtsuki T Apichartpiyakul C Uchiumi H Nojima Y Hoshino H (2009) Broad usage spectrum of G protein-coupled receptors as coreceptors by primary isolates of HIV. AIDS 23:761–769. 19307942 10.1097/QAD.0b013e328326cc0d

[B108] Skrzeczyńska-Moncznik J Stefańska A Zabel BA Kapińska-Mrowiecka M Butcher EC Cichy J (2009 a) Chemerin and the recruitment of NK cells to diseased skin. Acta Biochim Pol 56:355–360. 19543554 PMC8548436

[B109] Skrzeczyńska-Moncznik J Wawro K Stefańska A Oleszycka E Kulig P Zabel BA Sułkowski M Kapińska-Mrowiecka M Czubak-Macugowska M Butcher EC (2009 b) Potential role of chemerin in recruitment of plasmacytoid dendritic cells to diseased skin. Biochem Biophys Res Commun 380:323–327. 19168032 10.1016/j.bbrc.2009.01.071 PMC6289204

[B110] Southern C Cook JM Neetoo-Isseljee Z Taylor DL Kettleborough CA Merritt A Bassoni DL Raab WJ Quinn E Wehrman TS (2013) Screening β-arrestin recruitment for the identification of natural ligands for orphan G-protein-coupled receptors. J Biomol Screen 18:599–609. 23396314 10.1177/1087057113475480

[B111] Spiroglou SG Kostopoulos CG Varakis JN Papadaki HH (2010) Adipokines in periaortic and epicardial adipose tissue: differential expression and relation to atherosclerosis. J Atheroscler Thromb 17:115–130. 20145358 10.5551/jat.1735

[B112] Stefanov T Blüher M Vekova A Bonova I Tzvetkov S Kurktschiev D Temelkova-Kurktschiev T (2014) Circulating chemerin decreases in response to a combined strength and endurance training. Endocrine 45:382–391. 23783366 10.1007/s12020-013-0003-2

[B113] Stejskal D Karpisek M Hanulova Z Svestak M (2008) Chemerin is an independent marker of the metabolic syndrome in a Caucasian population--a pilot study. Biomed Pap Med Fac Univ Palacky Olomouc Czech Repub 152:217–221. 19219210 10.5507/bp.2008.033

[B114] Suzuki Y Haga S Katoh D So KH Choi KC Jung US Lee HG Katoh K Roh SG (2015) Chemerin is a novel regulator of lactogenesis in bovine mammary epithelial cells. Biochem Biophys Res Commun 466:283–288. 26342800 10.1016/j.bbrc.2015.08.105

[B115] Suzuki Y Hong YH Song SH Ardiyanti A Kato D So KH Katoh K Roh SG (2012) The regulation of chemerin and CMKLR1 genes expression by TNF-α, adiponectin, and chemerin analog in bovine differentiated adipocytes. Asian-Australas J Anim Sci 25:1316–1321. 25049696 10.5713/ajas.2012.12083 PMC4092937

[B116] Takahashi M Okimura Y Iguchi G Nishizawa H Yamamoto M Suda K Kitazawa R Fujimoto W Takahashi K Zolotaryov FN (2011) Chemerin regulates β-cell function in mice. Sci Rep 1:123. 22355640 10.1038/srep00123 PMC3216604

[B117] Takahashi M Takahashi Y Takahashi K Zolotaryov FN Hong KS Kitazawa R Iida K Okimura Y Kaji H Kitazawa S (2008) Chemerin enhances insulin signaling and potentiates insulin-stimulated glucose uptake in 3T3-L1 adipocytes. FEBS Lett 582:573–578. 18242188 10.1016/j.febslet.2008.01.023

[B118] Tang M Huang C Wang YF Ren PG Chen L Xiao TX Wang BB Pan YF Tsang BK Zabel BA (2016) CMKLR1 deficiency maintains ovarian steroid production in mice treated chronically with dihydrotestosterone. Sci Rep 6:21328. 26893072 10.1038/srep21328 PMC4759558

[B119] Tokizawa S Shimizu N Hui-Yu L Deyu F Haraguchi Y Oite T Hoshino H (2000) Infection of mesangial cells with HIV and SIV: identification of GPR1 as a coreceptor. Kidney Int 58:607–617. 10916084 10.1046/j.1523-1755.2000.00207.x

[B120] Vanhoutte PM Humphrey PP Spedding M (1996) X. International Union of Pharmacology recommendations for nomenclature of new receptor subtypes. Pharmacol Rev 48:1–2. 8685244

[B121] Vassilatis DK Hohmann JG Zeng H Li F Ranchalis JE Mortrud MT Brown A Rodriguez SS Weller JR Wright AC (2003) The G protein-coupled receptor repertoires of human and mouse. Proc Natl Acad Sci USA 100:4903–4908. 12679517 10.1073/pnas.0230374100 PMC153653

[B122] Vermi W Riboldi E Wittamer V Gentili F Luini W Marrelli S Vecchi A Franssen JD Communi D Massardi L (2005) Role of ChemR23 in directing the migration of myeloid and plasmacytoid dendritic cells to lymphoid organs and inflamed skin. J Exp Med 201:509–515. 15728234 10.1084/jem.20041310 PMC2213064

[B123] Wang C Wu WK Liu X To KF Chen GG Yu J Ng EK (2014) Increased serum chemerin level promotes cellular invasiveness in gastric cancer: a clinical and experimental study. Peptides 51:131–138. 24274970 10.1016/j.peptides.2013.10.009

[B124] Wang D Yuan GY Wang XZ Jia J Di LL Yang L Chen X Qian FF Chen JJ (2013) Plasma chemerin level in metabolic syndrome. Genet Mol Res 12:5986–5991. 24338392 10.4238/2013.November.26.8

[B125] Wang Q Kim JY Xue K Liu JY Leader A Tsang BK (2012) Chemerin, a novel regulator of follicular steroidogenesis and its potential involvement in polycystic ovarian syndrome. Endocrinology 153:5600–5611. 22948218 10.1210/en.2012-1424

[B126] Wanninger J Bauer S Eisinger K Weiss TS Walter R Hellerbrand C Schäffler A Higuchi A Walsh K Buechler C (2012) Adiponectin upregulates hepatocyte CMKLR1 which is reduced in human fatty liver. Mol Cell Endocrinol 349:248–254. 22118966 10.1016/j.mce.2011.10.032 PMC3670424

[B127] Wargent ET Zaibi MS O’Dowd JF Cawthorne MA Wang SJ Arch JR Stocker CJ (2015) Evidence from studies in rodents and in isolated adipocytes that agonists of the chemerin receptor CMKLR1 may be beneficial in the treatment of type 2 diabetes. PeerJ 3:e753. 25699203 10.7717/peerj.753 PMC4327305

[B128] Watts SW Dorrance AM Penfold ME Rourke JL Sinal CJ Seitz B Sullivan TJ Charvat TT Thompson JM Burnett R (2013) Chemerin connects fat to arterial contraction. Arterioscler Thromb Vasc Biol 33:1320–1328. 23559624 10.1161/ATVBAHA.113.301476 PMC3752465

[B129] Wittamer V Franssen JD Vulcano M Mirjolet JF Le Poul E Migeotte I Brézillon S Tyldesley R Blanpain C Detheux M (2003) Specific recruitment of antigen-presenting cells by chemerin, a novel processed ligand from human inflammatory fluids. J Exp Med 198:977–985. 14530373 10.1084/jem.20030382 PMC2194212

[B130] Wittamer V Grégoire F Robberecht P Vassart G Communi D Parmentier M (2004) The C-terminal nonapeptide of mature chemerin activates the chemerin receptor with low nanomolar potency. J Biol Chem 279:9956–9962. 14701797 10.1074/jbc.M313016200

[B131] Wu C Orozco C Boyer J Leglise M Goodale J Batalov S Hodge CL Haase J Janes J Huss JW III (2009) BioGPS: an extensible and customizable portal for querying and organizing gene annotation resources. Genome Biol 10:R130. 19919682 10.1186/gb-2009-10-11-r130 PMC3091323

[B132] Wu X Ye Y Rosell R Amos CI Stewart DJ Hildebrandt MA Roth JA Minna JD Gu J Lin J (2011) Genome-wide association study of survival in non-small cell lung cancer patients receiving platinum-based chemotherapy. J Natl Cancer Inst 103:817–825. 21483023 10.1093/jnci/djr075 PMC3096796

[B133] Xiaotao L Xiaoxia Z Yue X Liye W (2012) Serum chemerin levels are associated with the presence and extent of coronary artery disease. Coron Artery Dis 23:412–416. 22828724 10.1097/MCA.0b013e3283576a60

[B134] Yamaguchi Y Du XY Zhao L Morser J Leung LL (2011) Proteolytic cleavage of chemerin protein is necessary for activation to the active form, Chem157S, which functions as a signaling molecule in glioblastoma. J Biol Chem 286:39510–39519. 21949124 10.1074/jbc.M111.258921 PMC3234774

[B135] Yang M Yang G Dong J Liu Y Zong H Liu H Boden G Li L (2010) Elevated plasma levels of chemerin in newly diagnosed type 2 diabetes mellitus with hypertension. J Investig Med 58:883–886. 10.231/JIM.0b013e3181ec5db2 20601896

[B136] Yang YL Ren LR Sun LF Huang C Xiao TX Wang BB Chen J Zabel BA Ren P Zhang JV (2016) The role of GPR1 signaling in mice corpus luteum. J Endocrinol 230:55–65. 27149986 10.1530/JOE-15-0521 PMC5064765

[B137] Yoshimura T Oppenheim JJ (2011) Chemokine-like receptor 1 (CMKLR1) and chemokine (C-C motif) receptor-like 2 (CCRL2); two multifunctional receptors with unusual properties. Exp Cell Res 317:674–684. 21056554 10.1016/j.yexcr.2010.10.023 PMC3049852

[B138] Zabel BA Allen SJ Kulig P Allen JA Cichy J Handel TM Butcher EC (2005 a) Chemerin activation by serine proteases of the coagulation, fibrinolytic, and inflammatory cascades. J Biol Chem 280:34661–34666. 16096270 10.1074/jbc.M504868200

[B139] Zabel BA Kwitniewski M Banas M Zabieglo K Murzyn K Cichy J (2014) Chemerin regulation and role in host defense. Am J Clin Exp Immunol 3:1–19. 24660117 PMC3960757

[B140] Zabel BA Nakae S Zúñiga L Kim JY Ohyama T Alt C Pan J Suto H Soler D Allen SJ (2008) Mast cell-expressed orphan receptor CCRL2 binds chemerin and is required for optimal induction of IgE-mediated passive cutaneous anaphylaxis. J Exp Med 205:2207–2220. 18794339 10.1084/jem.20080300 PMC2556791

[B141] Zabel BA Silverio AM Butcher EC (2005 b) Chemokine-like receptor 1 expression and chemerin-directed chemotaxis distinguish plasmacytoid from myeloid dendritic cells in human blood. J Immunol 174:244–251. 15611246 10.4049/jimmunol.174.1.244

[B142] Zabel BA Zuniga L Ohyama T Allen SJ Cichy J Handel TM Butcher EC (2006) Chemoattractants, extracellular proteases, and the integrated host defense response. Exp Hematol 34:1021–1032. 16863908 10.1016/j.exphem.2006.05.003

[B143] Zhang R Liu S Guo B Chang L Li Y (2014) Chemerin induces insulin resistance in rat cardiomyocytes in part through the ERK1/2 signaling pathway. Pharmacology 94:259–264. 25471554 10.1159/000369171

[B144] Zhao RJ Pan ZY Long CL Cui WY Zhang YF Wang H (2013) Stimulation of non-neuronal muscarinic receptors enhances chemerin/ChemR23 system in dysfunctional endothelial cells. Life Sci 92:10–16. 23154239 10.1016/j.lfs.2012.10.029

[B145] Zhou J-X Liao D Zhang S Cheng N He HQ Ye RD (2014) Chemerin C9 peptide induces receptor internalization through a clathrin-independent pathway. Acta Pharmacol Sin 35:653–663. 24658352 10.1038/aps.2013.198 PMC4075970

